# Individual humans are more attractive to certain mosquito species

**DOI:** 10.1016/j.isci.2026.117006

**Published:** 2026-07-31

**Authors:** Kaylee M. Marrero, John S. Castillo, Dani Lucas-Barbosa, Anthony J. Bellantuono, Matthew A. Marrero, Dariel Cid, Andre L. Costa-da-Silva, Niels O. Verhulst, Matthew DeGennaro

**Affiliations:** 1 Biomolecular Sciences Institute, Florida International University, Miami, FL, USA; 2 Department of Biological Sciences, Florida International University, Miami, FL, USA; 3 National Centre for Vector Entomology, Institute of Parasitology, One Health Institute, Vetsuisse and Medical Faculty, University of Zürich, Zürich, Switzerland; 4 Institute of Environment, Florida International University, Miami, FL, USA

**Keywords:** mosquito, host attraction, human skin microbiome, human odor profiles, *Aedes aegypti*, *Aedes albopictus*, *Culex quinquefasciatus*, sex preference, host preference

## Abstract

Humans are not equally attractive to mosquitoes, leaving some more vulnerable to mosquito-borne illnesses than others. Body odor differences likely allow mosquitoes to discriminate between humans. Using a uniport olfactometer, we measured the attraction of *Aedes aegypti*, *Aedesalbopictus*, and *Culex quinquefasciatus* mosquitoes for each of our 119 participants. *Ae. aegypti*, but not other species tested, were slightly more attracted to male than female participants. Each of our three species ranked our participants differently, favoring a distinct subset of our cohort. For each species, mosquito attraction rates were used to define high- and low-attraction human odors and bacterial taxa. For example, *Ae. aegypti* and *Cx. quinquefasciatus* attraction was associated with the absence of odors like cyclic alcohols and monoterpenes, while *Ae. albopictus* attraction was associated with the presence of ketones. Each mosquito species exhibited distinct responses to individual humans, emphasizing both unique and shared cues for targeting their hosts.

## Highlights

• Mosquito species can rank the attractiveness of individual humans differently
• *Aedes aegypti* were slightly more attracted to male than female participants
• Attractive human odors and skin microbes differed for each mosquito species tested
• *Aedes* and *Culex* mosquitoes likely evolved distinct ways to sense humans

## Graphical abstract

**Figure:**
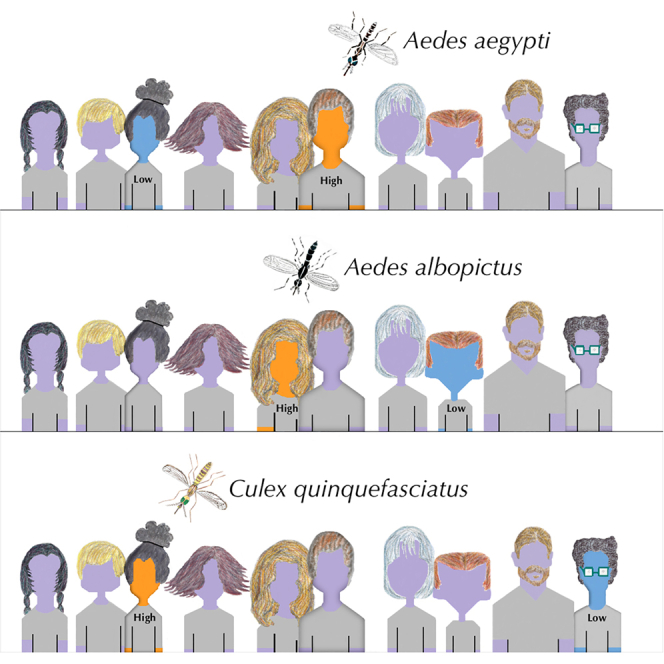

## Teaser

Biological Sciences; Zoology; Entomology; Biochemistry

## Introduction

Feeding on human blood is integral for the life cycle of highly anthropophilic mosquitoes such as *Aedes aegypti* and plays a significant role in species with a broader range of hosts such as *Culex quinquefasciatus*. The behavior of *Ae. aegypti* and *Aedes* *albopictus* female mosquitoes spread flaviviruses that cause dengue, Zika, and yellow fever, whereas *Cx. quinquefasciatus* can transmit the West Nile virus.^1^^,^^2^^,^^3^ In the Americas, all three mosquito species are predicted to increase their range, putting new populations at risk.^4^ Mosquitoes use a multimodal approach to find a suitable host, integrating the detection of carbon dioxide (CO_2_), body odor, visual cues, humidity, and heat.^5^^,^^6^^,^^7^^,^^8^^,^^9^^,^^10^^,^^11^^,^^12^ Anthropophilic mosquitoes prefer urbanized environments and are highly attracted to human scent, which varies considerably from person to person.^13^^,^^14^^,^^15^^,^^16^ Mosquitoes have evolved to use odor to guide them to their preferred hosts.^17^^,^^18^^,^^19^^,^^20^ Still, human scent is a complex odor space comprising over 1,000 volatile organic compounds (VOCs), many uncharacterized, making it difficult to determine which odor plume components are responsible for mosquito attraction.^21^^,^^22^^,^^23^^,^^24^^,^^25^ Isolating the components of human odor that attract or repel mosquitoes could lead to novel strategies to combat vector-borne illness.

Human odor is largely derived from the biotransformation of generally odorless skin secretions into VOCs by cutaneous bacteria of the human skin microbiome.^26^^,^^27^^,^^28^^,^^29^ The human skin microbiome is a relatively stable landscape of many different bacterial groups that utilize our sweat, sebum, and the stratum corneum as resources.^30^^,^^31^ Salient odors for mosquitoes may also be generated by UV light catalysis of sebum.^32^ Dominant taxa on our skin such as *Cutibacterium* spp.*, Staphylococcus* spp.*,* and *Corynebacterium* spp. produce odors that contribute to an individual’s signature scent that can attract mosquitoes.^33^^,^^34^^,^^35^^,^^36^ Not only are the human skin microbiomes of individuals stable over time, but the diversity of species is correlated with health and pregnancy, signaling the importance of the immune system in maintaining healthy populations of skin bacteria.^34^^,^^37^ Mosquito attraction to humans is thus directly related to our skin microbiome, and individuals who are favored likely have a higher mosquito bite risk.

As humans encounter mosquito species with a range of anthropophilic behavior, it is important to understand the common cues that underlie their attraction as well as the species-specific cues. Through the lens of a single mosquito species, previous work has explored the connection between mosquito attraction rates across human hosts and their microbiome or volatilomes.^38^^,^^39^^,^^40^^,^^41^^,^^42^^,^^43^^,^^44^^,^^45^^,^^46^ It has become increasingly clear that there is a connection between human skin microbes and/or odors when mosquitoes target individual humans.^28^^,^^40^^,^^41^^,^^42^^,^^43^^,^^44^^,^^45^^,^^46^^,^^47^^,^^48^^,^^49^^,^^50^ Although previous studies have compared the attraction of different mosquito species with individuals, cross-comparing attraction rates of multiple mosquito species to individual human volatilomes and skin microbiomes, to our knowledge, has not been previously accomplished.^21^^,^^51^ Using a group of 119 participants, we determined attraction rates of three vector mosquito species to each individual in our study. We found that *Ae. aegypti* were slightly more attracted to males than females, but no sex bias was detected in *Ae. albopictus* or *Cx. quinquefasciatus*. We also found that different mosquito species are usually not highly attracted to the same participants. Odors and bacterial taxa were associated with high- and low-attraction cohorts for each species tested. Here, we identify molecular signatures of mosquito attraction to inform next-generation repellent design and serve as indicators of species-specific mosquito bite risk.

## Results

### *Ae. aegypti* shows slightly increased attraction for self-reported males over females

**Figure 1:**
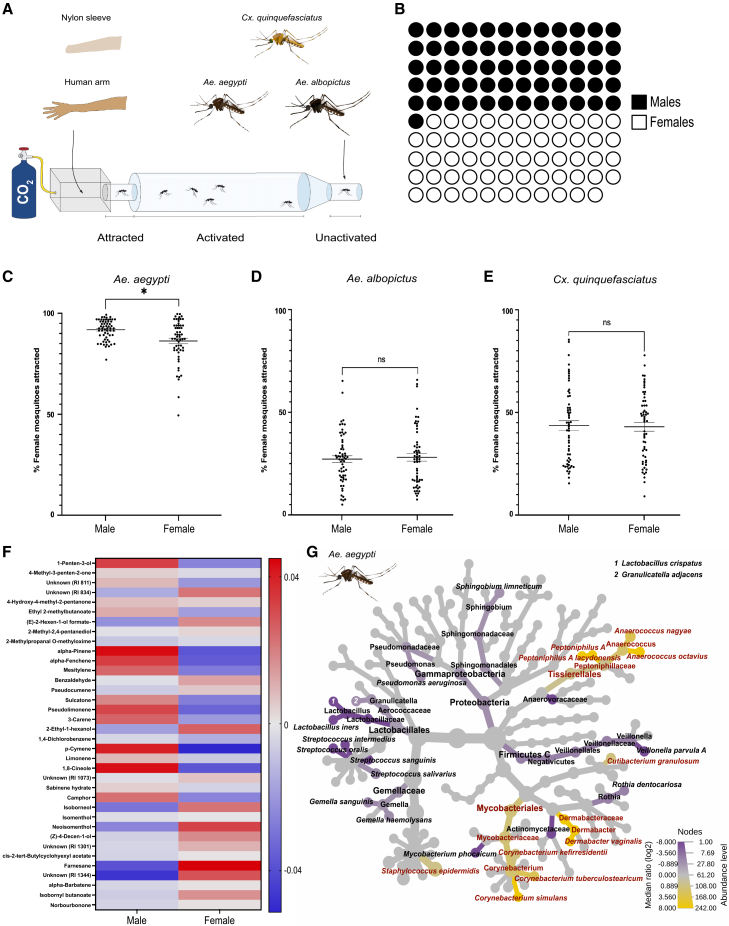
*Ae. aegypti* are more attracted to male than female participants (A) Schematic of the uniport olfactometer used for quantification of mosquito attraction to individual human arms for *Ae. aegypti* and *Ae. albopictus* or human-worn nylon sleeves in *Cx. quinquefasciatus* experiments. (B) Distribution of self-reported sex for the participants (*n* = 61 males and 58 females). (C–E) Wilcoxon rank-sum test of average mosquito attraction trials comparing all self-reported males and females in our study. Mean = 91.5 for *Ae. aegypti* males and 87.4 for females, *p* = 0.0204. Mean = 26.8 for *Ae. albopictus* males and 27.5 for females; *p* = 0.9998. Mean = 44.2 for *Cx. quinquefasciatus* males and 42.3 for females, *p* = 0.9997. Error bars represent standard deviation. ns indicates a *p* value ab 0.05. (F) GC-MS heatmap of chemical headspace for self-reported males (left) and females (right). Legend indicates the mean normalized peak count. (G) Phylogenetic heat tree exhibiting a Wilcoxon rank-sum test of bacterial species abundance. Labeled nodes represent taxa that are significantly abundant with an FDR-adjusted *p* value cutoff of 0.05. Yellow nodes represent increased abundance in male participants (dark red text), while purple nodes represent increased abundance in female participants (black text). Nodes labeled 1 and 2 (white text) are described in the upper right corner of this panel.

We recruited and tested 119 human participants from Miami, Florida. In addition to a demographically diverse group of volunteers, we studied the attraction rates of three different mosquito species, *Ae. aegypti, Ae. albopictus,* and *Cx. quinquefasciatus,* to these participants (Figures 1A and S1). To measure this behavior for *Ae. aegypti* and *Ae. albopictus*, participants placed their left forearm inside a uniport olfactometer with female mosquitoes on the opposite side of the apparatus.^52^^,^^53^ Since *Cx. quinquefasciatus* are night-biting mosquitoes, a nylon sleeve was used to capture the odors of each participant’s arms and experiments were conducted under nighttime lighting conditions in the uniport olfactometer.^41^^,^^42^^,^^43^^,^^44^ Each participant was tested in live host assays during their visit using different cohorts of *Ae. aegypti* or *Ae. albopictus* mosquitoes (see STAR Methods). At the same visit, participants donated nylon sleeves with their odor that were frozen for subsequent testing for *Cx. quinquefasciatus* attraction in the uniport olfactometer. Odor and microbiome samples were also collected during the participant’s visit. This approach allowed us to capture all relevant metrics used in this study on the same day, avoiding potential changes that could occur over time.

We calculated relative attraction as the number of mosquitoes that entered the attraction trap divided by the total (*n* = 30). *Ae. aegypti* showed an average attraction of 89% across all participants tested (Figure S2A), whereas *Ae. albopictus* exhibited the lowest overall attraction of 27% (Figure S2B). *Cx. quinquefasciatus* had an average attraction of 43% to human participant odor trapped on nylon sleeves (Figure S2C). To assess any effects of day of testing, we performed temporal autocorrelation testing. These tests indicated that for *Ae. aegypti* (Durbin-Watson test, *p* = 0.55), *Ae. albopictus* (Durbin-Watson test, *p* = 0.38), and *Cx. quinquefasciatus* (Durbin-Watson test, *p* = 0.6), there was no correlation between day of testing and mosquito behavioral responses to participants in the study. This suggests that the potential variation of the experimental conditions over time cannot explain the differences in mosquito attraction we found.

To begin assessing attraction differences throughout the entire group, we analyzed the mosquito behavior, volatilome, and microbiome results of all self-reported male (*n* = 61) and female participants (*n* = 58) in our study (Figure 1B). We compared the attraction rates for *Ae. aegypti, Ae. albopictus,* and *Cx. quinquefasciatus* and found that *Ae. aegypti* showed a slight but significantly higher attraction for male participants (*p* = 0.0204, mean for males = 91.5, and mean for females = 87.4) (Figure 1C), whereas less anthropophilic species in our study did not show a difference in attraction between males and females (Figures 1D and 1E; mean for 1D males = 26.8 and mean for 1D females = 27.5; mean for 1E males = 44.2 and mean for 1E females = 42.3). Additionally, to evaluate whether demographics significantly affected mosquito behavioral responses, a linear mixed model (LMM) was constructed for each of the three vector species using age, race, and sex, as well as body temperature as fixed covariates. Only the *Ae. aegypti* attraction rate LMM showed sex (Male) was significant (*p* value <0.05, Table S1). For *Ae. albopictus* and *Cx. quinquefasciatus*, none of the covariates were significant (*p* value < 0.05, Table S1). When the participant’s race was reported as Black, White, or mixed, the results were also significant for *Ae. aegypti* (*p* value <0.05, Table S1); we note that the Cohen’s d values for the demographics of our cohort are not consistent across demographic comparisons, suggesting that our study is underpowered to address the question of race/ethnicity in mosquito attraction^51^^,^^52^ (Table S2).

To ascertain the specific human odors that each mosquito species detects, the dynamic headspace from the left arm of each participant was collected. VOCs were identified by extracting volatiles from the left arm of participants, which was enclosed in a nylon bag for 90 minutes immediately after undergoing mosquito behavior experiments. Using gas chromatography-mass spectrometry (GC-MS), we analyzed the volatiles from the headspace of a human arm with k-means clustering into six different groups (Figure S3). We found males exhibited a higher abundance of the alcohol, 1-penten-3-ol, and the terpenes, alpha-pinene, alpha-fenchene, p-cymene, limonene, and pseudolimonene, compared with females (Figure 1F). For females, the terpene farnesane, the terpene derivative, isoborneol, and the cyclic alcohol, neoisomenthol were more abundant on their skin compared with males (Figure 1F).

Next, we compared the human skin microbiomes of males and females to evaluate the bacterial species that are associated with this difference in *Ae. aegypti* attraction. We sampled from the antecubital fossa and proximal volar forearm to explore the relationship of the arm with host-seeking variability in these vectors. We performed full-length 16S ribosome sequencing to identify bacteria comprising each participant’s human skin microbiome. Using a Wilcoxon rank-sum heat tree based on abundance level and median log ratio, we found several species that were significantly more abundant in males when compared with females, including *Anaerococcus nagyae*, *A. octavius*, *Co. kefirresidentii*, *Co. simulans*, *Co. tuberculostearicum*, *Cu. granulosum*, *Dermabacter vaginalis*, and *Staphylococcus epidermidis* (Wilcoxon test, FDR-adjusted *p* < 0.05) (Figure 1G and Data S1). We found that two amplicon sequence variants (ASVs) of *Cu. acnes* were detected in all males, while only one ASV of *Cu. acnes* was detected in all females. The second ASV of *Cu. acnes* was detected in all female participants except one (Figure S4). Although we detected sex differences in skin microbiota and odor profiles that correlate with *Ae. aegypti* attraction, further studies are required to determine how important a human’s sex is when this mosquito species is choosing an individual to target.

### Individual humans are more attractive to certain mosquito species

**Figure 2:**
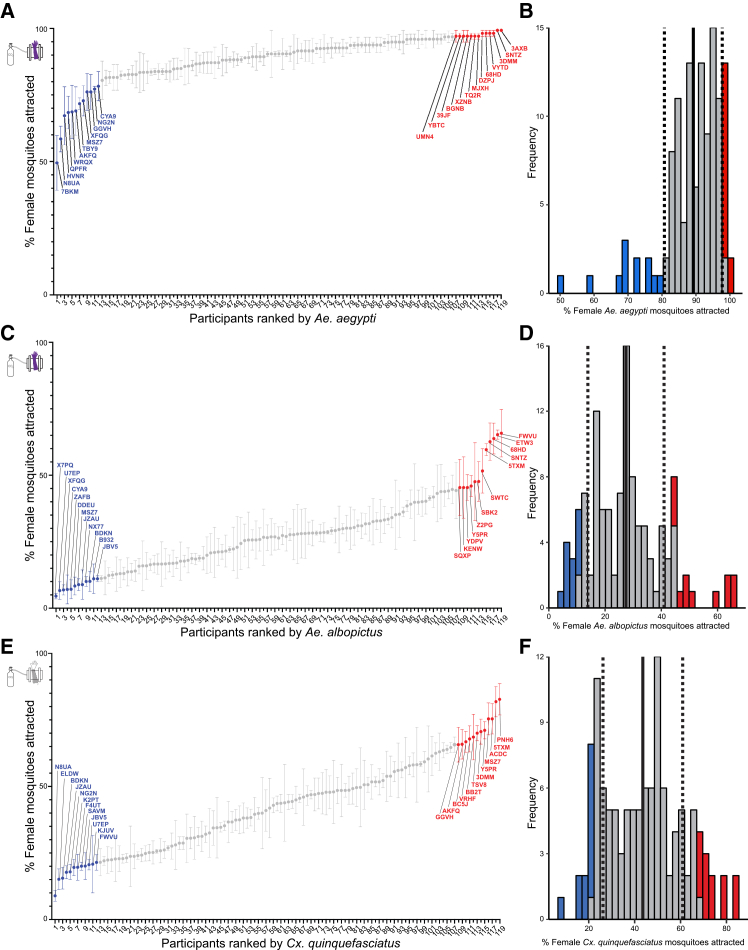
Distribution of participants by mosquito species attraction rates (A) Average attraction rates of female *Ae. aegypti* mosquitoes to individual humans. Participants are represented by a number ranked by ascending attraction to *Ae. aegypti*. Red dots reflect high-attraction participants, while blue dots represent low-attraction participants. Error bars represent standard deviation. (B) Histograms of all participants’ average attraction rates to *Ae. aegypti*. Red bars are data from the high-attraction cohorts, and blue bars are data from the low-attraction cohorts. Solid lines in on the graph indicate the mean of the dataset, while dotted lines show standard deviations. (C) Average attraction rates of female *Ae. albopictus* mosquitoes to individual humans. Red dots reflect high-attraction participants, while blue dots represent low-attraction participants. Error bars represent standard deviation. (D) Histograms of all participants’ average attraction rates to *Ae. albopictus.* (E) Average attraction rates of female *Cx. quinquefasciatus* mosquitoes to human odor trapped on nylon sleeves. Red dots reflect high-attraction participants, while blue dots represent low-attraction participants. Error bars represent standard deviation. (F) Histograms of all participants’ average attraction rates to *Cx. quinquefasciatus.*

Given the number of odors and bacteria that differ between our male and female participants, it is difficult to discern which of these are the salient cues for *Ae. aegypti*. In addition, we wanted to test whether participants were similarly attractive to our three mosquito species or whether there was species-specific variation. To address this, we identified high- and low-attraction participants for *Ae. aegypti*, *Ae. albopictus*, and *Cx. quinquefasciatus*. High- and low-attraction participants were separated for each species using a percentile analysis to isolate participants in the top 10% (90^th^ percentile) and bottom 10% (10^th^ percentile) of ranked mean attraction. This yielded 13 high-attraction participants and 12 low-attraction participants for *Ae. aegypti* (Figures 2A and 2B)*,* 12 high and low-attraction participants for *Ae. albopictus* (Figures 2C and 2D), and 12 high- and low-attraction participants for *Cx. quinquefasciatus* (Figures 2E and 2F). A Wilcoxon test showed that there were significant differences in attraction between the high- and low-attraction groups for each species (Figure S5 and Data S2) (*p* < 0.001). We also wanted to assess any differences in attraction that could have been caused by the time of the visit (i.e., morning or afternoon). We did not find any significant differences for *Ae. aegypti* or *Ae. albopictus* attraction for our live host tests (Figures S6A and S6B) Given that *Cx. quinquefasciatus* behavior was tested with nylon sleeves which the participant was told to wear for 12 to 16 h, the length of time the participant wore the sleeve could be potentially affected by the time of day the participant arrived for the study visit. However, no bias was found between sleeves collected in the morning or the afternoon (Wilcoxon test, *p* = 0.33, Figure S6C).

For each mosquito species, we generated histogram distributions of the attraction data and found that all participants in the high- and low-attraction groups were at least one standard deviation apart from the mean (Figures 2B, 2D, and 2F). We determined that median coefficient of variation (CV) of attraction to individual participants for each species. *Ae. aegypti* was low at 5.39%. *Cx. quinquefasciatus* and *Ae. albopictus* showed higher median CVs of 19.98% and 34.64%, respectively. However, a reduction in median CV is seen for all species in high-attraction participants when compared to all subjects (*Ae. aegypti* HA 1.97% vs. 5.39% for all participants; *Cx. quinquefasciatus* HA 12.49% vs. 19.98% for all participants; *Ae. albopictus* HA 21.47% vs. 34.64% for all participants). In contrast, an increase in median CV is seen for all species in low-attraction participants when compared to all participants (*Ae. aegypti* LA 15.30% vs. 5.39% for all participants; *Cx. quinquefasciatus* LA 33.92% vs. 19.98% for all participants; *Ae. albopictus* LA 67.71% vs. 34.64% for all participants). Taken together, these differences may suggest that an individual’s cues can increase or reduce the variation in how a mosquito responds to them. We caution that species-specific differences in standard deviation require further investigation to determine their biological relevance.

**Figure 3:**
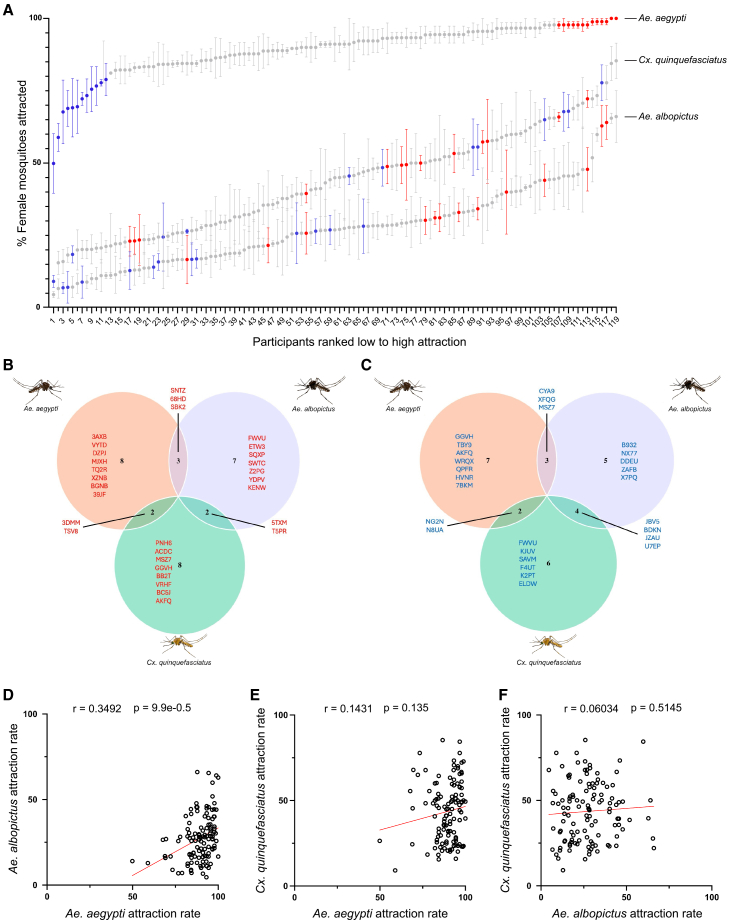
Individual humans are more attractive to certain species of mosquitoes (A) Average behavioral responses in ascending order for all 119 participants to the three mosquito species studied. Order of participants on the *x* axis is unchanged from Figure 2; however, red dots reflect *Ae. aegypti* high-attraction participants, whereas blue dots represent *Ae. aegypti* low-attraction participants. High- and low-attraction groups were determined by percentile analysis for the greatest and lowest 10% of participants. Error bars represent standard deviation. (B and C) Venn diagrams of all high-attraction participants for each species (B) and low-attraction participants for each species (C). (D–F) Pearson’s r correlation scatterplots of mosquito species ranked attraction to all participants. Each point represents each participant’s attraction rate for *Ae. aegypti* (D and E), or *Ae. albopictus* (F) on the *x* axis and attraction rate for *Ae. albopictus* (D) or *Cx. quinquefasciatus* (E and F) on the *y* axis.

When overlaying all three behavioral plots without changing the participant order (lowest to highest attraction), we found that the participants who were most and least attractive for *Ae. aegypti* were often ranked differently by *Ae. albopictus* and *Cx. quinquefasciatus* (Figure 3A). Some participants shared high attraction to more than one mosquito species, but no participants had high attraction for all three mosquito species (Figure 3B). Similarly, some participants shared low-attraction across more than one mosquito species, but no participants had low attraction to all three mosquito species (Figure 3C). Looking at all 119 participants, individual human attraction rates of *Ae. aegypti* and *Ae. albopictus* were weakly correlated (Pearson’s r = 0.3492) (Figure 3D). The correlation between individual human attraction rates of *Ae. aegypti* and *Cx. quinquefasciatus* of all participants was negligible (Pearson’s r = 0.1431) (Figure 3E). A similar result was observed for *Ae. albopictus* and *Cx. quinquefasciatus* (Pearson’s r = 0.06034) (Figure 3F). Taken together, this suggests that each mosquito species prefers a different cohort of individuals.

### Participant volatilomes are associated with species-specific mosquito responses

**Figure 4:**
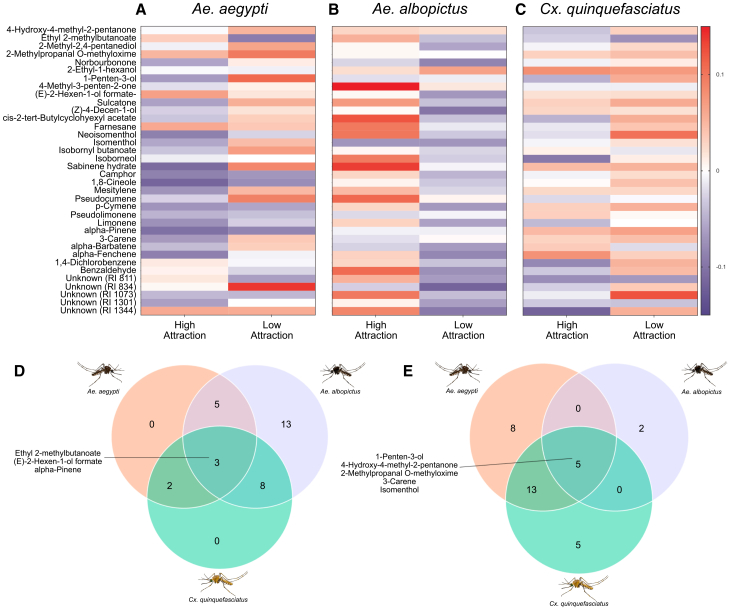
Distinct odor repertoires of individual humans are associated with species-specific mosquito attraction (A–C) GC-MS heatmap of chemical headspace for high (left) and low (right) attraction to (A) *Ae. aegypti,* (B) *Ae. albopictus,* or (C) *Cx. quinquefasciatus* mosquitoes. Values listed represent the average chemical peak area. Legend indicates an increase or decrease in the average chemical peak area. (D) Venn diagram of compounds that are more prevalent in high-attraction participants than low-attraction for all mosquito species. (E) Venn diagram of compounds that are more prevalent in low-attraction participants than high attraction for all mosquito species.

Dynamic headspace analysis resulted in the identification of VOCs associated with each mosquito species. We found that only ethyl 2-methylbutanoate is more abundant in the high-attraction group for *Ae. aegypti* than the low-attraction group (Figure 4A). Reduced attraction of *Ae. aegypti* was uniquely associated with higher levels of eight volatiles found in low-attraction participants, with no species-specific odors associated with attraction identified (Table S2). The converse was true for *Ae. albopictus*; 13 species-specific volatiles were more prevalent in high-attraction participants than in low-attraction participants. Among these was sulcatone, which has been linked to the evolution of mosquito preference for humans.^18^^,^^54^ Only 2-ethyl-1-hexanol was species-specifically elevated in the *Ae. albopictus* low-attraction group (Figure 4B). Alpha-barbatene, alpha-fenchene, (E)-2-hexen-1-ol formate, (Z)-4-decen-1-ol, and pseudolimonene concentrations were elevated in participants who were highly attractive to *Cx. quinquefasciatus* (Figure 4C). Limonene was more abundant in the headspace of low-attraction participants for this species, whereas mesitylene abundance was the same in both high- and low-attraction participants (Figure 4C). Both compounds are worthy of further investigation as potential influencers of *Culex* species behavior. Interestingly, ethyl 2-methylbutanoate, (E)-2-hexen-1-ol formate, and alpha-pinene were all more prevalent in high-attraction participants for all three vector species (Figure 4D). Similarly, 1-penten-3-ol, 4-hydroxy-4-methyl-2-pentanone, 2-methylpropanal *O*-methyloxime, 3-carene, and isomenthol were all more prevalent in low-attraction participants for all three species (Figure 4E). Taken together, our results indicate that the presence of volatiles associated with low attraction in *Ae. aegypti* and *Cx. quinquefasciatus* may drive mosquito decisions to target individual humans. In contrast, *Ae. albopictus* may be guided to favored individuals by attractive odor cues (Figures 4D and 4E).

### Mosquito species are attracted to individuals with different skin microbial communities

**Figure 5:**
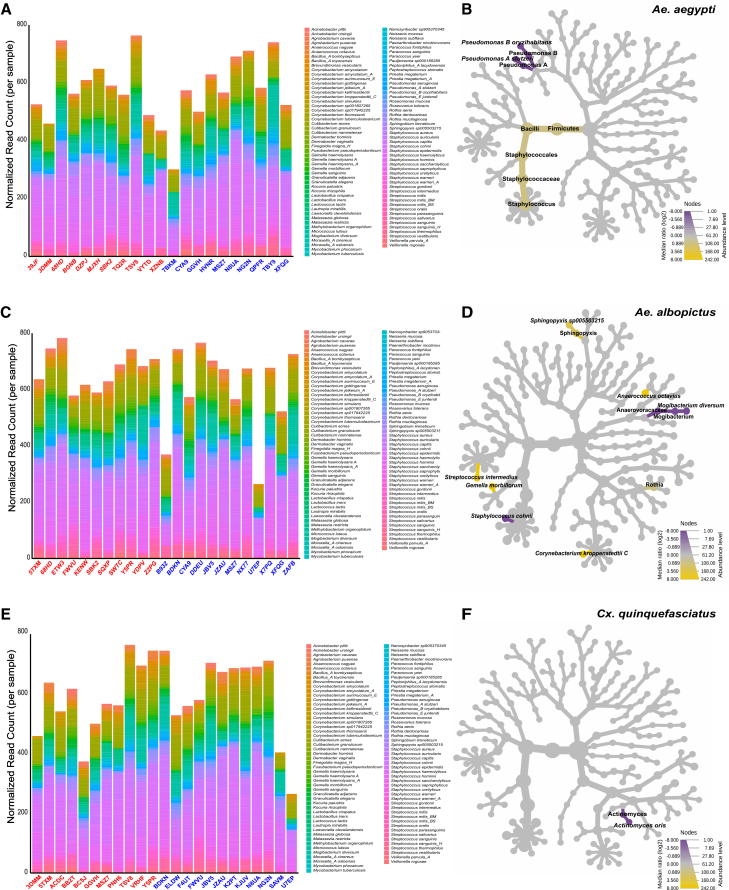
Mosquito species attraction rates are associated with distinct taxa of human skin bacteria (A, C, and E) Stacked bar chart depicting abundance of skin commensals on the arm of high- and low-attraction participants to (A) *Ae. aegypti,* (C) *Ae. albopictus,* or (E) *Cx. quinquefasciatus.* Bacteria are colored by species. (B, D, and F) Phylogenetic heat tree exhibiting a Wilcoxon rank-sum test of bacterial species abundance. Yellow nodes represent abundance in high-attraction participants in (B) *Ae. aegypti,* (D) *Ae. albopictus,* or (F) *Cx. quinquefasciatus,* whereas purple nodes represent abundance in low-attraction participants. Labeled nodes represent taxa that are significantly abundant with an FDR-adjusted *p* value cutoff of 0.05.

We examined the abundance and the diversity of the bacterial taxa present on the skin of our participants. We identified 246 distinct bacterial taxa across our 119 participants, and only one of these was shared by all participants (Data S3). This indicates that differences in bacterial taxa could explain differences in mosquito attraction rates between individuals. For example, the skin microbiome for *Ae. aegypti* high- and low-attraction groups showed large amounts of *Staphylococcus* and *Corynebacterium* spp.*,* which are largely found across body sites^55^^,^^56^ (Figure 5A). We found that the Firmicutes phylum, Bacilli class, Staphylococcales order, Staphylococcaceae family, and *Staphylococcus* genus were enriched in participants who are highly attractive to *Ae. aegypti* mosquitoes. This is consistent with previous literature linking attraction of *Ae. aegypti* and Anopheles *gambiae* mosquitoes to *Staphylococcus* bacteria.^38^^,^^40^^,^^48^^,^^57^ For low-attraction participants, *Pseudomonas A* and *Pseudomonas B* genera, which differ based on the structure of the flagella, as well as *Pseudomonas A stutzeri* and *Pseudomonas B oryzhibitans* were more abundant^58^ (Wilcoxon test, FDR-adjusted *p* < 0.05) (Figure 5B and Data S4). For sister species *Ae. albopictus*, we saw that the normalized read count was higher on average across the high-attraction group than those of *Ae. aegypti* and *Cx. quinquefasciatus* (Figures 5C and S7). We found *Sphingopyxis* spp., *Sphingopyxis* sp005503215, *A. octavius*, *Rothia* spp., *Co. kroppenstedtii C*, and *Streptococcus intermedius* were significantly associated with the high-attraction group whereas *Staphylococcus cohnii,* Anaerovaracaceae family, *Mogibacterium* spp.*,* and *Mogibacterium diversum* were significantly abundant in low-attraction participants (Wilcoxon test, FDR-adjusted *p* < 0.05) (Figure 5D and Data S5). Finally, for *Cx. quinquefasciatus* mosquitoes, we found that the normalized read count was lower on average for both the high- and low-attraction groups than that of *Ae. aegypti* and *Ae. albopictus* (Figures 5E and S7). No bacterial species abundance was significantly associated with high attraction to *Cx. quinquefasciatus*, but *Actinomyces* spp. and *Actinomyces oris* were enriched in the low-attraction group (Wilcoxon test, FDR-adjusted *p* < 0.05) (Figure 5F and Data S6). We found each mosquito species’ attraction rates are associated with participants who possess distinct bacterial signatures.

**Figure 6:**
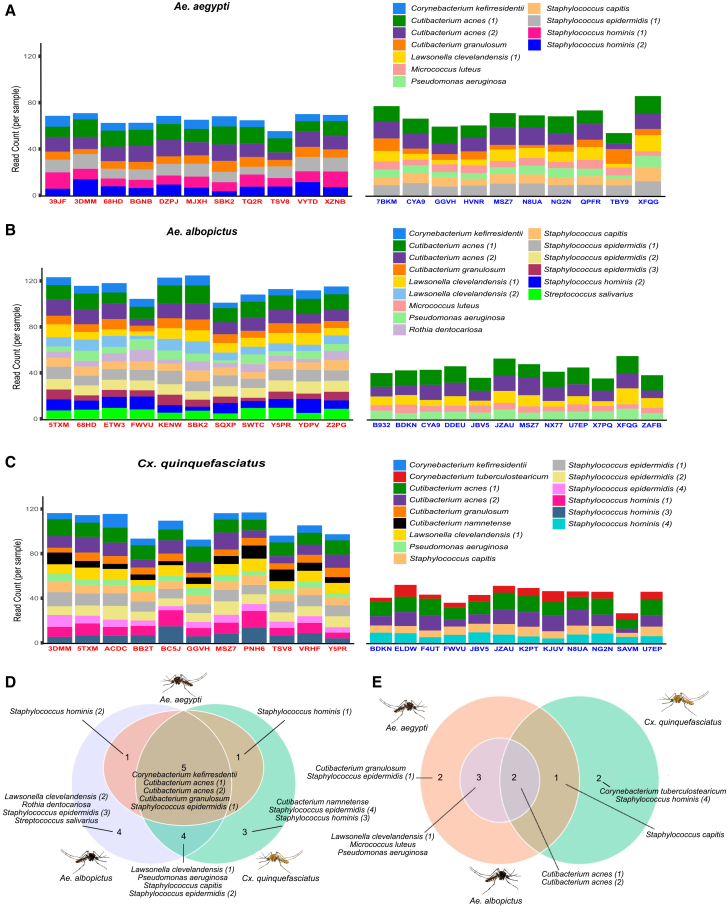
Core microbial communities reveal distinct and common taxa associated with species-specific mosquito attraction (A–C) Stacked bar chart depicting the core skin commensals present for all high (left) or low (right) attraction participants for (A) *Ae. aegypti,* (B) *Ae. albopictus,* or (C) *Cx. quinquefasciatus.* Bacteria are labeled by ASV. (D) Venn diagram of core microbes for all high-attraction participants across all mosquito species. (E) Venn diagram of core microbes for all low-attraction participants across all mosquito species.

We identified skin bacteria shared by participants in high- or low-attraction groups classified by mosquito species. Bacteria that were present for every participant of a high- or low-attraction group were assessed as the core microbiome for a given mosquito species. There were seven bacteria detected in all *Ae. aegypti* high-attraction participants and eight bacteria were detected in all *Ae. aegypti* low-attraction participants (Figure 6A). Some bacteria overlapped with the high-attraction group, including both *Cu. acnes* variants, *Cu. granulosum,* and the same ASV of *S. epidermidis*; however, other bacteria were distinct for the low-attraction core microbiome, such as *Micrococcus luteus* and *S. capitis.* Interestingly, *P. aeruginosa* was not detected in the high-attraction core microbiome but was detected in the low-attraction core microbiome. The core microbiome for *Ae. albopictus* high-attraction participants had 14 total bacteria and 5 in the low-attraction group. The low-attraction group also included the two ASVs of *Cu. acnes, Micrococcus luteus,* and *P. aeruginosa* (Figure 6B)*.* Notably, no *Staphylococcus* spp. appears in this low-attraction core microbiome. Finally, 13 bacteria strains were detected in all *Cx. quinquefasciatus* high-attraction participants, including the two ASVs of *Cu. acnes, Cu. granulosum, P. aeruginosa,* three ASVs of *S. epidermidis,* and two variants of *S. hominis* (Figure 6C). The low-attraction core microbiome contained five species, including two ASVs of *Cu. acnes, S. capitis,* and one variant of *S. hominis*. This was the only group to contain *Co. tuberculostearicum* in any core microbiomes.

We did not detect any distinguishing core microbes in *Ae. aegypti* high-attraction participant from other species tested, while *Ae. albopictus* high-attraction participants had four distinct bacterial species and *Cx. quinquefasciatus* high-attraction participants had three distinct bacterial species. There were only five bacteria that were associated with high attraction across all three mosquito species: *Co. kefirresdentii, Cu. acnes* (1), *Cu. acnes* (2), *Cu. granulosum,* and *S. epidermidis* (1) (Figure 6D). No unique core microbes were detected in low-attraction *Ae. albopictus* participants, but two distinct bacteria were detected in each of the *Ae. aegypti* and *Cx. quinquefasciatus* low-attraction participants. Only two ASVs of *Cu. acnes* comprised the shared core microbiome for low-attraction participants across all vector species tested (Figure 6E). Across *Ae. aegypti*, *Ae. albopictus*, and *Cx. quinquefasciatus* mosquitoes, we detected three core bacteria in common for high-attraction participants that were not found across the low-attraction core microbiomes. These bacterial taxa, *Co. kefirresidentii*, *Cu. granulosum*, and *S. epidermidis,* may broadly signal to mosquitoes the presence of an attractive host.

## Discussion

To determine species-specific odor and bacterial signatures that drive mosquito attraction to humans, we tested the attraction of 119 participants to three mosquito vector species: *Ae. aegypti, Ae. albopictus,* and *Cx. quinquefasciatus*. Our findings indicated sex-specific differences in *Ae. aegypti* mosquito attraction but not in *Ae. albopictus* or *Cx. quinquefasciatus* mosquitoes*.* When we analyzed the odor profiles and microbiomes between male and female participants, we found many differences that may contribute to *Ae. aegypti* attraction rates. There was high diversity in individual skin microbiomes of the participants in our study. Strikingly, out of the 246 bacterial taxa detected, the core microbiomes of all our participants shared only one species of bacteria.

Male participants exhibited a significant increase in bacteria associated with the production of fatty acid volatiles, including the *Corynebacterium* genus, that may enhance the attraction of *Ae. aegypti*.^59^^,^^60^ Notably, we found female participants showed higher abundances of *Pseudomonas aeruginosa,* which is consistent with previous work depicting a reduced attraction in both *An. gambiae* and *Ae. aegypti* mosquitoes.^48^^,^^49^ Although we have identified many differences between male and female odor plumes and skin bacteria, we do not know which of these features make male participants more attractive than female participants to *Ae. aegypti* mosquitoes. Given this complexity, we sought to identify the cues that correlate with attraction levels for each of the three vector species.

We found that different odors contributed to each mosquito species’ attraction rates to humans. High attraction of *Ae. aegypti* and *Cx. quinquefasciatus* was associated with lower abundance of 13 odors in the collected headspace. For *Ae. aegypti,* only one odor was more abundant in high-attraction participants than low-attraction participants, and there were no odors detected in these high-attraction participants that distinguished them from the other species tested. Yet, eight compounds are species-specific for the *Ae. aegypti* low-attraction cohort (alpha-fenchene, mesitylene, p-cymene, sabinene hydrate, (Z)-4-decen-1-ol, unknown RI 1301, alpha-barbatene, and isobornyl butanoate). *Ae. aegypti* is almost exclusively anthropophilic, so we expect the olfactory receptors of these mosquitoes to be finely tuned for specific human cues and perhaps odors that do not signal “human” could reduce host-seeking.^6^^,^^15^^,^^18^^,^^61^ *Cx. quinquefasciatus* followed a similar pattern to *Ae. aegypti* and did not have any species-specific odors for high attraction but had five for low attraction (farnesane, isoborneol, 1,4-dichlorobenzene, an unknown odor RI 811, and benzaldehyde). Behaviorally, these mosquitoes are more opportunistic and will blood feed on birds, pigs, and reptiles as well as humans.^62^^,^^63^^,^^64^ The association of high-attraction participants with the absence of odors in *Cx. quinquefasciatus* suggests that these odors, some of which have been linked with repellency, allow this species to distinguish hosts that contain a broad set of potential attractants with a narrower set of potentially repellent compounds. Our evidence suggests the absence of key odors may drive the decision of *Ae. aegypti* or *Cx. quinquefasciatus* mosquitoes to target a human host. Further testing of these odors is needed to determine if they are repellents.

Conversely, we found that *Ae. albopictus* attraction rates were associated with the presence of attractive odors. Along with strong preferences for cyclic alcohols and terpenoids, we found that *Ae. albopictus* high-attraction cohort had 13 species-specific odors that were not found in the other three species tested. Several of these odors are derived from plant-like environments and may reflect the generalist feeding behavior and ecological flexibility of *Ae. albopictus*.^65^^,^^66^ *Ae. albopictus* have been shown to be opportunistic in their choice of hosts.^67^^,^^68^^,^^69^^,^^70^ The cues that *Ae. albopictus* use for host-seeking are more closely related to ancestral, zoophilic *Ae. aegypti formosus* rather than the human-specialized *Ae. aegypti aegypti* that inhabit urbanized environments.^71^ Therefore, it is likely to be evolving its own distinct path to human host detection, and our results support that *Ae. albopictus’* attraction is guided by a different set of odors than its sister species *Ae. aegypti*.^72^^,^^73^^,^^74^

Both abundance and community composition of the human skin microbiome contribute to species-specific mosquito attraction via odor production.^75^^,^^76^^,^^77^ Biotransformation of the products of sweat and sebum produce VOCs, but community interactions also play a role in the resulting odor plume.^29^^,^^36^^,^^55^^,^^56^^,^^78^^,^^79^^,^^80^ For *Ae. aegypti* and *Ae. albopictus,* attraction may be driven by both the presence and absence of attractive and repellent bacterial taxa. Conversely, attraction rates of *Cx. quinquefasciatus* mosquitoes may be driven by the absence of repellent bacterial taxa. Unsurprisingly, *Pseudomonas* spp. was detected not only in higher abundance in *Ae. aegypti* low-attraction participants, but also in the low-attraction core microbiome. We have identified several species of *Pseudomonas* that are associated with low attraction for *Ae. aegypti*, including *Pseudomonas A stutzeri*, *Pseudomonas B oryzihabitans*, and *Pseudomonas aeruginosa.* This may indicate that *Pseudomonas* spp. is responsible for the production of repellent odors. In studies with *An. gambiae*, *Pseudomonas aeruginosa* has been associated with individuals that are poorly attractive.^38^ For both *An. gambiae* and *Ae. aegypti*, *in vitro* microbial communities with higher ratios of *Pseudomonas aeruginosa* were less attractive.^38^^,^^49^ Additionally, we hypothesize that *Pseudomonas* spp. exhibit antimicrobial activity within the skin microbiome community, which could explain both its higher abundance and contribution to the odor plume.^81^^,^^82^^,^^83^ *Cx. quinquefasciatus* has only one microbe that is significantly more abundant in the low-attraction skin microbiome, *Actinomyces oris*. This species has been used as antimicrobial larvicide in vector control strategies, so it may share similar interspecific bacterial interactions amongst the skin microbiome.^84^^,^^85^^,^^86^

For *Ae. albopictus,* we hypothesize that the human skin bacteria are driving attraction by producing more attractive odors. We found that there were twice as many significantly abundant microbial species for high-attraction participants than low-attraction participants. Of interest was the *Anaerococcus* spp.*,* including *A. octavius,* which is linked to the production of volatile fatty acids through the breakdown of sweat components.^87^ It is likely that biotransformations such as these lead to attractive odor plumes that *Ae. albopictus* mosquitoes can detect.

How humans are generally perceived by mosquitoes is becoming clearer, but what determines whether an individual is targeted by a mosquito has been difficult to assess.^6^^,^^7^^,^^11^^,^^18^^,^^41^^,^^88^^,^^89^^,^^90^ We have found that species-specific odor and bacteria signatures associated with individual humans are correlated with attraction rates for female *Ae. aegypti, Ae. albopictus,* and *Cx. quinquefasciatus* mosquitoes. This suggests that different mosquito species are attracted to different human cues. Of the 3,500 mosquito species, only a few have developed anthropophily, and the evolutionary paths to this are likely to be distinct.^91^ Our dataset provides perspective on how attraction to humans in three vector species can be driven by both distinct and common cues and is consistent with each species independently evolving its host preference(s). Unraveling this complexity will aid ongoing efforts to generate microbial-based mosquito repellents that produce repulsive compounds or reduce attractive cues.^92^ The bacterial and odor signatures provided may also be useful to interrupt mosquito interactions with humans.

### Limitations of the study

In using the uniport olfactometer, we are presenting the attraction of mosquitoes to individual humans; we are not directly assessing whether a given mosquito will select an individual out of a group. Even though we attempted to regulate the experimental conditions for uniformity, mosquito species may be differentially sensitive to microscale environmental factors that could potentially affect behavior. The odor-trapped on nylon sleeves approach used in the *Cx. quinquefasciatus* behavior experiments have been supported as a reasonable approximation of attraction to live human arms in previous studies, but we recognize that they are not a direct replacement for live hosts. The increased standard deviation in participant trials and lower overall response rates in *Cx. quinquefasciatus* and *Ae. albopictus* when compared with *Ae. aegypti* may or may not be due to biologically relevant factors, and further laboratory and field studies are needed to clarify their range of responses to humans. Our study lays the groundwork for such an experimental investigation.

## Resource availability

### Lead contact

Further information and requests for resources and reagents should be directed to and will be fulfilled by the lead contact, Matthew DeGennaro (mdegenna@fiu.edu).

### Materials availability

Mosquito strains are available from the lead contact upon request.

### Data and code availability

• The underlying data for this study have been made publicly available through the Dryad Data repository: https://doi.org/10.5061/dryad.vdncjsz97 and the NCBI Sequence Read Archive: PRJNA1415315.
• All original code generated for this paper is publicly available through the Dryad Data repository: https://doi.org/10.5061/dryad.vdncjsz97.
• Any additional information required to reanalyze the data reported in this paper is available from the lead contact upon request.

## Acknowledgments

We would like to thank the DeGennaro lab for their support and feedback on the manuscript. We thank Kristian Lopez for his technical contributions to the manuscript. We also appreciate the suggestions Dr. Takeshi Morita and Dr. Sheng-Hao Lin provided for the manuscript. The following reagent was obtained through BEI Resources, NIAID, NIH: *Ae. albopictus* F39 Strain, MRA No. MRA-804 and *Cx. quinquefasciatus* JHB F172 Strain, MRA No. NR-43025. We thank Alejandro Acuna for creating the graphical abstract for our manuscript.

This work was supported by grants from 10.13039/100000185Defense Advanced Research Projects Agency (10.13039/100000185DARPA) 10.13039/100021538Biological Technologies Office (BTO) (HR0011-20-C-0073) (to J.S.C., A.J.B., D.C., A.L.C.S., N.O.V., M.D.); 10.13039/100000030Centers for Disease Control and Prevention (10.13039/100000030CDC), Southeastern Center of Excellence in Vector-borne Disease (U01CK000662) (to K.M.M.); and Florida International University Graduate Teaching (to M.A.M.).

## Author contributions

Conceptualization, N.O.V. and M.D.; methodology, J.S.C., K.M.M., D.L.B., A.J.B., D.C., A.L.C.-d.-S., N.O.V., and M.D.; software, K.M.M., A.J.B., and M.A.M.; formal analysis, K.M.M., J.S.C., D.L.B., A.J.B., and M.A.M.; investigation, J.S.C., K.M.M., D.L.B., A.J.B., D.C., M.D., and A.L.C.-d.-S.; validation, J.S.C., K.M.M., A.L.C.-d.-S., and M.D.; resources, N.O.V. and M.D.; data curation, J.S.C., K.M.M., A.L.C.-d.-S., and M.D.; writing – original draft, K.M.M., J.S.C., and M.D.; writing – review and editing, K.M.M., D.L.B., A.J.B., D.C., M.A.M., A.L.C.-d.-S., N.O.V., and M.D.; visualization, J.S.C., K.M.M., M.A.M., and M.D.; project administration, N.O.V. and M.D.; funding acquisition, N.O.V. and M.D.

## Declaration of interests

The authors declare no competing interests.

## STAR★Methods

### Key resources table

**Table:** 

REAGENT or RESOURCE	SOURCE	IDENTIFIER
**Chemicals, peptides, and recombinant proteins**		

Tris acetate, EDTA buffer	Sigma-Aldrich	T9650-4L
Tween 20	Sigma-Aldrich	CAS 9005-64-5
DNA/RNA Shield	Zymo Research	Cat. #R1100-250
Defibrinated sheep blood	Remel Inc, Thermo Fisher Scientific	#R54020
Adenosine triphosphate	Thermo Fisher Scientific	#34369-07-8

**Critical commercial assays**		

ZymoBIOMICS DNA Microprep Kit	Zymo Reserach	Cat. #D4305
KAPA HiFi HotStart ReadyMix PCR Kit	KAPA Biosystems	Cat. # KK2602

**Deposited data**		

Data from this study for Figures 1, 2, 3, 4, 5, and 6 as well as all supplementary figures, tables, and data	Dryad	https://doi.org/10.5061/dryad.vdncjsz97
DNA sequence files	NCBI Sequence Read Archive	[NCBI SRA]: [PRJNA1415315]

**Experimental models: Organisms/strains**		

*Ae. aegypti:* Orlando	Matthew DeGennaro	Vosshall Laboratory
*Ae. albopictus:* F39 strain	USDA	MRA No. MRA-804
*Cx. quinquefasciatus*: JHB F172 strain	USDA	MRA No. NR-43025

**Oligonucleotides**		

27F Primer	ZiomoBIOMICS Microrep Kit	5′GCATC/barcode/AGRGTTYGATYMTGGCTCAG3′
1492R Primer	ZimoBIOMICS DNA Microprep Kit	5′GCATC/barcode/RGYTACCTTGTTACGACTT3′

**Software and algorithms**		

RStudo	R	v2023.06.0 + 421
GraphPad Prism	GraphPad	v9
Met-Align MSClust	See Lommen and Kools,^93^ Lommen,^94^ Tikunov^95^	v1.0.4
QIIME2	See Bolyen et al.^96^	v2019.1
MicrobiomeAnalyst	See Dhariwal et al.^97^	v2.0
Phyloseq	See McMurdie and Holmes^98^	v1.46.0
ggplot2	See Wickham^99^	v3.5.2
DHARMa	See Hartig^100^	v0.4.7
glmmTMB	See Brooks et al.^101^	v1.1.11
cAIC4	See Säfken et al.^102^	v1.1
VSEARCH	See Rognes et al.^103^	v2.30.6
Performance	See Lüdecke et al.^104^	v0.15.0
DADA2	See reference Callahan et al.^105^	v1.30.0
G∗Power	See Kang^106^	v3.1

**Other**		

TetraMin tropical fish foodperformance	Tetra	Cat. # 16152
Membrane feeders	Chemglass	Cat. # CG-1835-70

### Experimental model and study participant details

#### Statement of research ethics

All research conducted was in compliance with the NIH guidelines and the Florida International University Environmental Health and Safety guidelines. All research in this study was reviewed and approved by the Florida International University Institutional Review Board under protocol #IRB-16-0386-AM02.

#### Mosquito rearing

*Ae. aegypti, Ae. albopictus,* and *Cx. quinquefasciatus* mosquitoes were reared and maintained following the methodology outlined in David et al., 2023. In brief, *Ae. aegypti* mosquitoes were kept at 27 ± 1°C and 70 ± 10% humidity with a 14:10 light-dark cycle. Eggs were hatched in 0.5 L pre-boiled, deoxygenated water with a TetraMin food tablet in a sealed Mason jar (Tetra, Melle, Germany). 250 L2 larvae were reared in 2 L DI water with TetraMin tablets until pupation, then transferred to DI water in a ramekin inside a BugDorm cage (MegaView Science Co., Ltd., Taiwan). Adults were maintained at a 1:1 male:female ratio with 10% sucrose provided *ad libitum*. For egg production, 5–7-day-old females were blood-fed using a glass feeder filled with pre-warmed defibrinated sheep blood (#R54020, Remel Inc, Thermo Fisher Scientific, Lenexa, KS) and ATP (#34369-07-8, Thermo Fisher Scientific, Pittsburgh, PA), covered with Parafilm, and connected to a circulating water bath at 37°C. Feeding lasted approximately 1 h. Three days later, a filter paper-lined ramekin with DI water was placed in the cage for oviposition.

### Method details

#### Recruitment

Prior to recruitment, we performed a power analysis using the G∗Power software (v3.1) with alpha = 0.05 and power = 0.80 to calculate the number of male and female participants necessary to detect sex-related differences in attraction.^106^^,^^107^^,^^108^ Since effect sizes that are too small can be considered trivial even with statistical significance, we chose a medium effect size of 0.5. While our power analysis indicated that 102 total participants (51 male and 51 female) were necessary, we recruited more to ensure we could evaluate differences between these groups. To characterize the signatures of human odor that drive mosquito attraction, we recruited, obtained informed consent, and successfully assayed 119 participants between the ages of 18–59. Participants self-reported their race and ethnicity, with White Hispanic/Latinx participants making up 42%, 21% identified as White non-Hispanic, 16% as Black, 15% as Multiracial, and 6% as Asian. The sex of participants was also self-reported, with 51% male participants and 49% female participants.

#### Assessing the attractiveness of individual humans

Mosquitoes were placed in a custom-made uniport olfactometer to analyze their behavioral attraction.^8^^,^^52^ The uniport olfactometer consists of a large plexiglass tube (75 cm long and 13 cm wide) connected to a small cylindrical cage (13 cm long and 5 cm in diameter) that contains the mosquitoes prior to the experiment. At the far end of the plexiglass tube connected to the stimulus chamber, the left arm of the participant is inserted in an enclosed space with dimensions of 25 cm by 20 cm by 13 cm. In the stimulus chamber, carbon-filtered, humidified air and CO_2_ can combine with odorants to attract mosquitoes that have been released from a trap. Acrylic flowmeter Model VFA-4- SSV (Dwyer Instruments Inc., IN, USA) set to 3 SCFH was used to measure the CO_2_ release rate in the stimulus chamber. The final concentration of CO_2_ in the assay was maintained at 2500–2700 ppm by a carbon dioxide monitor (Catalog#CO2-100, Amprobe). At the same time, the airflow rate was set at 21 standard cubic feet per hour by an air flowmeter (King Instruments CA, USA). The sealed design of the uniport, air filtration, and the positive pressure caused by air circulation in the apparatus will isolate the assay from all possible environmental scents.

Prior to experimentation, participants were instructed to refrain from showering the night before their visit and were heavily discouraged from wearing skin products such as deodorants, antiperspirants, perfume, or cologne. Previous research in our lab indicated that *Ae. aegypti* mosquitoes would show approximately 15% attraction to carbon dioxide with air flow in the uniport olfactometer.^8^ Prior to testing each participant or nylon sleeve, a blank (no human or nylon sleeve) was run in the uniport olfactometer to test if there was any residual odor in the assay. If mosquito attraction was over 15%, each piece of the uniport olfactometer was cleaned, then the blank test was run again. This process was repeated until the mosquito response was under 15% to ensure there was no odor contamination before beginning any human or nylon sleeve behavior testing. Uniport olfactometry was performed during volunteer visits for both *Ae. aegypti* (Orlando strain) and *Ae. albopictus* (F39 strain, USDA, MRA No. MRA-804), in triplicate for each species, to determine attraction. To quantify *Cx. quinquefasciatus* (JHB F172 strain, USDA, MRA No. NR-43025) attraction, participants wore nylon stockings (brown L’eggs Everyday brand, Amazon) over their arms for 12-16 h to collect odor. The stockings were worn following their shower the evening before the study and were collected at the beginning of their visit and stored at −20°C until use in the olfactometer. Mosquitoes aged 7–21 days were used, obtained from 1 to 3 independent cages. All individuals were reared under standardized conditions, and age variation was restricted to avoid early or senescent stages.

Each olfactometer experiment was conducted for 8 min and contained 30 females of each species. Each participant underwent six trials of olfactometer assays, three with *Ae. aegypti* and three with *Ae. albopictus*. *Cx. quinquefasciatus* are night-biting mosquitoes, and trials were performed under moonlight (lux 0.05–0.1) conditions using nylon sleeves in the olfactometer rather than a live human. Previous studies have used human odor trapped on nylon sleeves or fabric to attract *Cx. quinquefasciatus*.^42^^,^^43^^,^^44^ During the investigation with participants, the mosquito species is randomized. All mosquitoes were subjected to behavioral experiments only once before being sacrificed.

Mosquito attraction rates were calculated as done previously.^53^ All assays were initially set up with 30 mosquitoes in the WHO tube release chamber. Mosquitoes that left the release chamber and entered the attraction trap were scored as attracted. Mosquitoes that did not leave the release chamber were scored as unactivated and included in the mosquito total. Mosquitoes that were dead were removed from the mosquito total. The attraction rate was calculated by dividing the number of attracted mosquitoes by the total number of live mosquitoes.

#### Profiling body odor collection

To identify volatile organic compounds (VOCs) emanating from the epidermis, we collected volatiles from the headspace of each participant’s left arm. The left arm was placed in a nylon bag (Toppits, Cofresco Frishhalteprodukte GmbH & Co., Minden, Germany), and their volatiles were collected for 90 min on Tenax adsorbent fibers by circulating ultra zero grade air (Airgas) at a rate of 400 mL min^−1^ per minute into the top of the bag and simultaneously applying a vacuum (GAST, Model DOA-P704-AA, Michigan, USA) to pull air at a 200 mL min^−1^ rate to the back of the stainless steel thermal desorption tubes filled with 200 mg of Tenax TA.

Using a GC-MS equipped with a thermodesorption unit, we characterized human volatile profiles. VOCs were desorbed from the Tenax tubes for 10 min at 250°C and captured in a sorbent trap chilled with liquid nitrogen at −110°C. Compounds were desorbed from this trap during secondary desorption at 40°C s^−1^ and desorbed at 280°C for 10 min, and later transferred in split mode to a non-polar gas-chromatography (GC) column (RXI-5ms 30 m × 0.25 mm×1.00 μm). The chromatographic procedure was conducted at a carrier gas flow rate of 1 mL min^−1^. The temperature of the GC oven was programmed to rise from 40°C (5 min hold time) to 280°C (8 min hold time) at a rate of 5°C min^−1^. The temperature of the MS transfer line was set to 280°C. The electron beam energy was set to 70 eV, and the ion source temperature was set to 250°C. The mass spectrometer scanned m/z 35–400 at a rate of 4.7 scans s^−1^. Helium gas was used for desorption and chromatographic analyses. GC-MS data were processed using the MetAlign–MSClust software pipeline.^93^ In brief, MetAlign corrects the baseline and eliminates the noise of each GC-MS output file. Subsequently, it aligns the individual mass peaks in all chromatograms.^94^ MSClust then clusters the aligned mass peaks so that mass spectra of putative compounds are reconstructed.^95^

#### Sampling the human skin microbiome

Following behavioral analysis, the participant rested their arm atop a table covered with a sterile surgical drape. A total of four samples were collected from the underside of the individual’s arm, each comprising a 5 × 5 cm area. The sampling sites are located on the distal volar forearm (beginning just above the wrist crease), mid-volar forearm, proximal volar forearm, and antecubital fossa.

For each sample site, two sterile double swabs (BD BB CultureSwab EZ II) were used to collect samples. Each swab was moistened with a sterile aliquot of SCF-1 buffer (50 mM Tris buffer [pH 7.6], 1 mM EDTA [pH 8.0], and 0.5% Tween 20) and vigorously swabbed against the skin for 2 min, rolling swabs to collect along the entire flocked surface. Swabs from each sample site were clipped with sterilized cutters into a 25 mL conical tube containing 1 mL of DNA/RNA Shield (Zymo Research, California, USA) and mixed. Blank samples were collected for each participant by waving swabs moistened with SCF-1 buffer in the air for 2 min, then collected and processed identically in DNA/RNA Shield.

For extraction, samples were first vortexed for 1 min to displace cells from the swab to the DNA/RNA Shield. The suspension was then transferred to a BashingBead Lysis Tube containing 0.1 & 0.5 mm glass beads (Zymo Research, California, USA). Sample suspensions were then lysed in the ZR BashingBead Lysis tube (0.1 & 0.5 mm beads) in DNA/RNA Shield using the Omni Bead Rupto-24 Elite (Omni International, Kennesaw, GA) (velocity of 6 m/s for 1 min, 5 min rest, cycle is repeated three times for a total of 3 min of bead beating). DNA was extracted from lysed samples using the ZymoBIOMICS DNA Microprep Kit (Zymo Research). DNA samples were amplified with indexed 27F (5′GCATC/barcode/AGRGTTYGATYMTGGCTCAG3′) and 1492R (5′GCATC/barcode/RGYTACCTTGTTACGACTT3′) primers, with 16 base pair barcodes used in unique combinations to identify libraries. Libraries were constructed in a 25 μL reaction volume with 2.5 μM of each primer and 12.5 μm KAPA HiFi HotStart ReadyMix PCR Kit (KAPA Biosystems). Libraries were then amplified using the following program: a 95°C initial denaturation for 3 min, followed by 28 cycles of amplification with 95°C denaturation for 30 s, 57°C annealing for 30 s, and 30-s extension at 72°C, with a final extension step at 72°C for 60 s. Libraries were purified on AMPure PB beads (Pacific Biosciences), quantified via Qubit dsDNA HS (Thermo Fisher), and normalized prior to pooling. SMRTbell libraries were constructed from amplicon pools and sequenced for a 15-h movie duration on the Sequel II Platform (Pacific Biosciences). Participant human skin microbiomes were characterized with full-length (V1-V9) 16S amplicon sequencing using PacBio HiFi sequencing. Previous mosquito/host microbiome investigations used short fragment Illumina 16S sequencing, however, our method captures all the hypervariable areas of 16S, vastly improving the assay’s taxonomic accuracy. Demultiplexed libraries were denoised to amplicon sequence variants (ASVs) using DADA2^105^ and analyzed using QIIME 2,^96^ with classification performed using VSEARCH^103^ in combination with the Silva 138 99% OTUs full-length sequences database. The ASV method begins by identifying which precise sequences were read and how often each precise sequence was read. These data are then combined with an error model for the sequencing run, allowing the comparison of similar readings to determine the probability that a particular read at a particular frequency is not due to sequencing error and subsequently filtered for PCR chimeras.^109^ Essentially, this generates a *p*-value for each exact sequence, where the null-hypothesis is equivalent to that exact sequence being the result of sequencing error.^105^ This resulted in the species-level identification of nearly all major taxa present in the samples. Occasionally, this method can identify fungal species via off-target sequencing or non-specific amplification of mitochondrial DNA, and so any detected fungal species were removed from the analysis.^110^^,^^111^^,^^112^

### Quantification and statistical analysis

#### Identifying high and low attraction participants

To assess if there were any temporal effects that could cause the mosquito to behave differently, we performed to analyses. To assess the effects of day of testing with mosquito behavior for all three species, we tested for temporal autocorrelation using the residuals of the linear mixed models. We used the *DHARMa* package^100^ in *R/R Studio* to first simulate the residuals, then recalculated these simulated residuals. This allowed the package to detect any patterns of temporal affecting the attraction data for each species as well as generating a DHARMa object. We performed a Durbin-Watson test for each of the three mosquitoes and found that all *p*-values were greater than 0.05, indicating no day-of-testing correlation with mosquito attraction for any of the three species. Second, we performed Wilcoxon tests to address if there was any difference in mosquito behavior between participants tested in the morning or the afternoon. Again, we did not find any significant differences between morning participants and afternoon participants for all three mosquito species.

High and low attraction participants for *Ae. aegypti, Ae. albopictus*, and *Cx. quinquefasciatus* were identified through a percentile analysis to isolate participants in the top 10% (90^th^ percentile) and bottom 10% (10^th^ percentile) of ranked attraction for each vector species. This gave us 13 high attraction participants and 12 low attraction participants for *Ae. aegypti,* 12 high and low attraction participants for *Ae. albopictus*, and 12 high and low attraction participants for *Cx. quinquefasciatus.* We generated histograms for We then compared both high and low attraction groups for each vector species with a Wilcoxon rank-sum test, and both groups significantly differed from each other (*p* < 0.0001).

#### Identification of odor profiles

After GC-MS output files were analyzed using the MetAlign-MSClust software, mass spectra data was normalized around the mean. Thiry-one odors were identified with only five unknown odors. Participants were then grouped by their self-reported sex or into their respective high and low attraction cohorts for *Ae. aegypti, Ae. albopictus,* and *Cx. quinquefasciatus.* Using these cohorts, the average chemical peak for each odor was calculated and plotted as a heatmap. Legend values represent the mean chemical peak reading. Further, they were set to have a midpoint of zero and boundary values were chosen to maximize visibility of the heatmap.

#### Identification of microbial components

The microbiome data were analyzed using the Quantitative Insights into Microbial Ecology 2 (QIIME2) (version 2019.1) program. The feature table, phylogeny data, and metadata table were then imported for analysis into MicrobiomeAnalyst (version 2.0).^97^ Participants 3AXB, RPYF, SNTZ, AKFQ, TAFA, and WRQX did not have microbiome sequencing data, so these participants were excluded from all microbiome analyses. Based on the mean abundance of operational taxonomical units (OTUs), samples were filtered for low prevalence (removed if 10% of counts were less than 4 across all samples) and 10% variability using a rank-based inter-quantile range assessment. The data was normalized using cumulative sum scaling (CSS). Stacked column graphs were constructed with this software using the 16S reads as relative abundance. The participants of each group made up each column (x axis), and total bacterial counts made the y axis. Stacks in the columns are constructed from the read counts for each OTU in the microbiome for the group. A Wilcoxon rank-sum heat tree was created to visualize differences between high attraction and low attraction groups for each vector species.

#### Core microbiomes for high and low attraction cohorts

The level of attraction to each of the three mosquito species (*Ae. aegypti, Ae. albopictus,* or *Cx. quinquefasciatus*) was matched with each participant’s microbiome sequencing data so that groups that are high attraction or low attraction to each species were formed. Using the *phyloseq* R package,^98^ physeq objects were created for each mosquito species by combining the feature table, phylogeny data, and metadata. Each of these physeq objects were filtered for a minimum of four read counts in 10% of the data and for a rank-sum based inter-quantile variance of 10%. Data were then scaled using cumulative sum scaling (CSS). Zeroes from the data were dropped, and the groups in the physeq object were separated by the metadata to form distinct, filtered, and scaled high- and low-attraction groups for each vector species. Next, each group was assessed for bacteria that are present across every participant in the group (e.g., bacteria that are present in all participants who are highly attractive to *Ae. aegypti*). These bacteria were classified at the amplicon sequence variant (ASV) level, and the scaled counts of reads were recorded. Finally, stacked column graphs were constructed using the *ggplot2* package,^99^ where the volunteers in each group made up each column (x axis), and the total microbial counts made the y axis. Stacks in the columns are constructed from the read counts for each ASV in the core microbiome for the group. All data analysis was performed using *R/RStudio*.

#### Core microbiomes for male and female participants

Participants were matched with their reported biological sex and their microbiome sequencing data so that male and female groups were formed. Using the *phyloseq* R package, physeq objects were created for each mosquito species by combining the feature table, phylogeny data, and metadata. Each of these physeq objects were filtered for a minimum of four read counts in 10% of the data and for a rank-sum based inter-quantile variance of 10%. Data was then scaled using cumulative sum scaling (CSS). Zeroes from the data were dropped, and the groups in the physeq object were separated by the metadata to form distinct, filtered, and scaled male and female groups. Next, each group was assessed for bacteria that were present across every participant in the group (e.g., bacteria that were present in every male in the study). These bacteria were classified at the amplicon sequence variant (ASV) level, and the scaled counts of reads were recorded. Finally, stacked column graphs were constructed using the *ggplot2* package, where the volunteers in each group made up each column (x axis), and the total bacterial counts made the y axis. Stacks in the columns are constructed from the read counts for each ASV in the core microbiome for the group. All data analysis was performed using *R/RStudio*.

#### Linear mixed model analysis

We used a general linear mixed model (LMM) to evaluate the effect of demographic variables on the percentage of mosquitoes attracted to our volunteers for each species of mosquito. Here, we use subject ID as the random effects variable to control for any intrinsic human factors affecting baseline attraction. We began with a full LMM including all variables using the *glmmTMB* package in *R,*^101^ then removed the least significant variable in a stepwise method of model selection. Prior to running each model, we check for any correlation between the continuous variables. We used the *performance*^104^ and *DHARMa* packages to examine the variance inflation factor (VIF) in addition to other variables. Variables with a high VIF (>10) were removed from the LMM prior to the next iteration. The most optimized model was chosen based on the conditional Akaike Information Criterion (cAIC) of the model summary using the *cAIC4* package.^102^ We then selected the simplest model iteration within 2 cAIC change of the most complex model. This process was repeated for each of the three mosquito species.
