# Supplementary material for: Individual humans are more attractive to certain mosquito species
Source: iScience. 2026 Jul 31;29(8):117006. doi: 10.1016/j.isci.2026.117006 (PMC13453020; doi:10.1016/j.isci.2026.117006)
Supplement: Supplementary file 1 [file mmc1.pdf]

iScience, Volume *29*

Supplemental information

Individual humans are more attractive to certain mosquito species

Kaylee M. Marrero, John S. Castillo, Dani Lucas-Barbosa, Anthony J. Bellantuono, Matthew A. Marrero, Dariel Cid, Andre L. Costa-da-Silva, Niels O. Verhulst, and Matthew DeGennaro

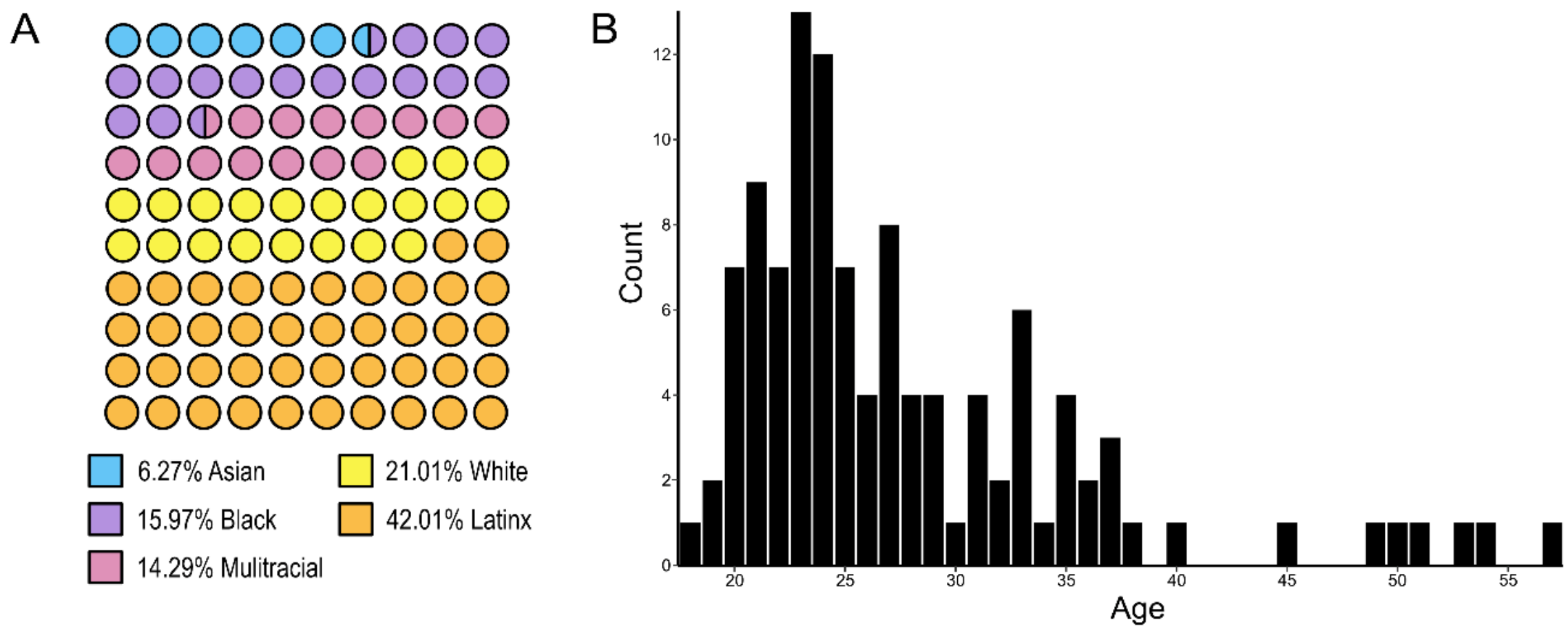

Figure S1. (A) Distribution of self-reported race for the 119 participants in this study. **(B)** Histogram showing age distributions of the participants in the study.

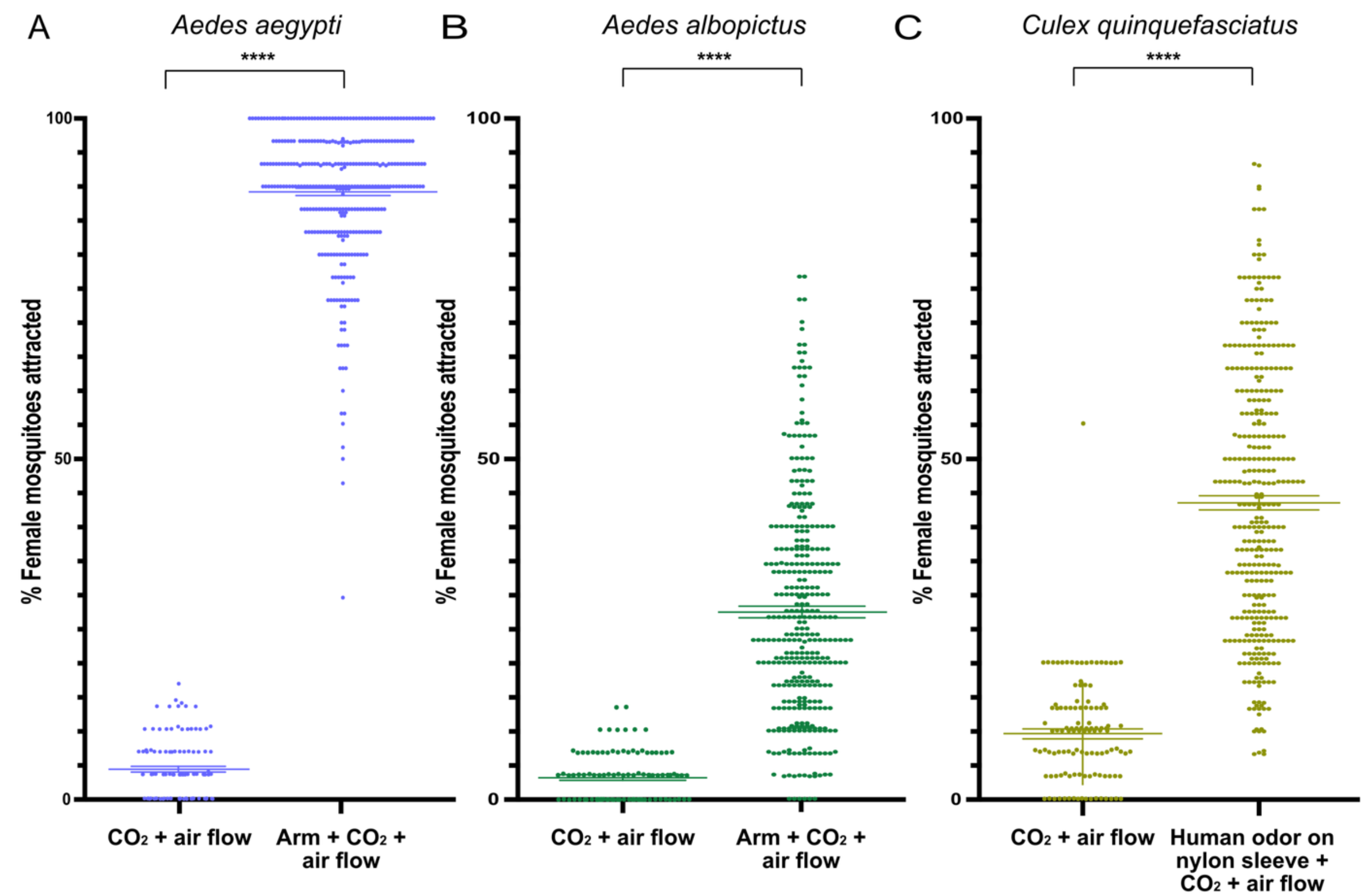

Figure S2. Overall attraction rates of **(A)** *Aedes aegypti,* **(B)** *Aedes albopictus,* and **(C)** *Culex quinquefasciatus* female mosquitoes for each uniport olfactometer experiment. The y-axis represents the percentage of female mosquitoes attracted per trial. Data points on the right for each species show individual behavior run values for either the participant's arm (A&B) or the human odor on nylon sleeve (C). Horizontal lines indicate the standard error of the mean. Data points on the left for each species show blank run values using only carbon dioxide and air flow. Depicted are all data points from blanks that were recorded to test for residual odor contamination. For any one blank run that was greater than 15% attraction under these conditions, the uniport olfactometer was cleaned completely and reevaluated until attraction was below 15%. Horizontal lines indicate the standard error of the mean. For all three species, blank run values were significantly different than behavior run values (Wilcox test, $P < 2.2\text{x}10^{-16}$). See Methods for additional details.

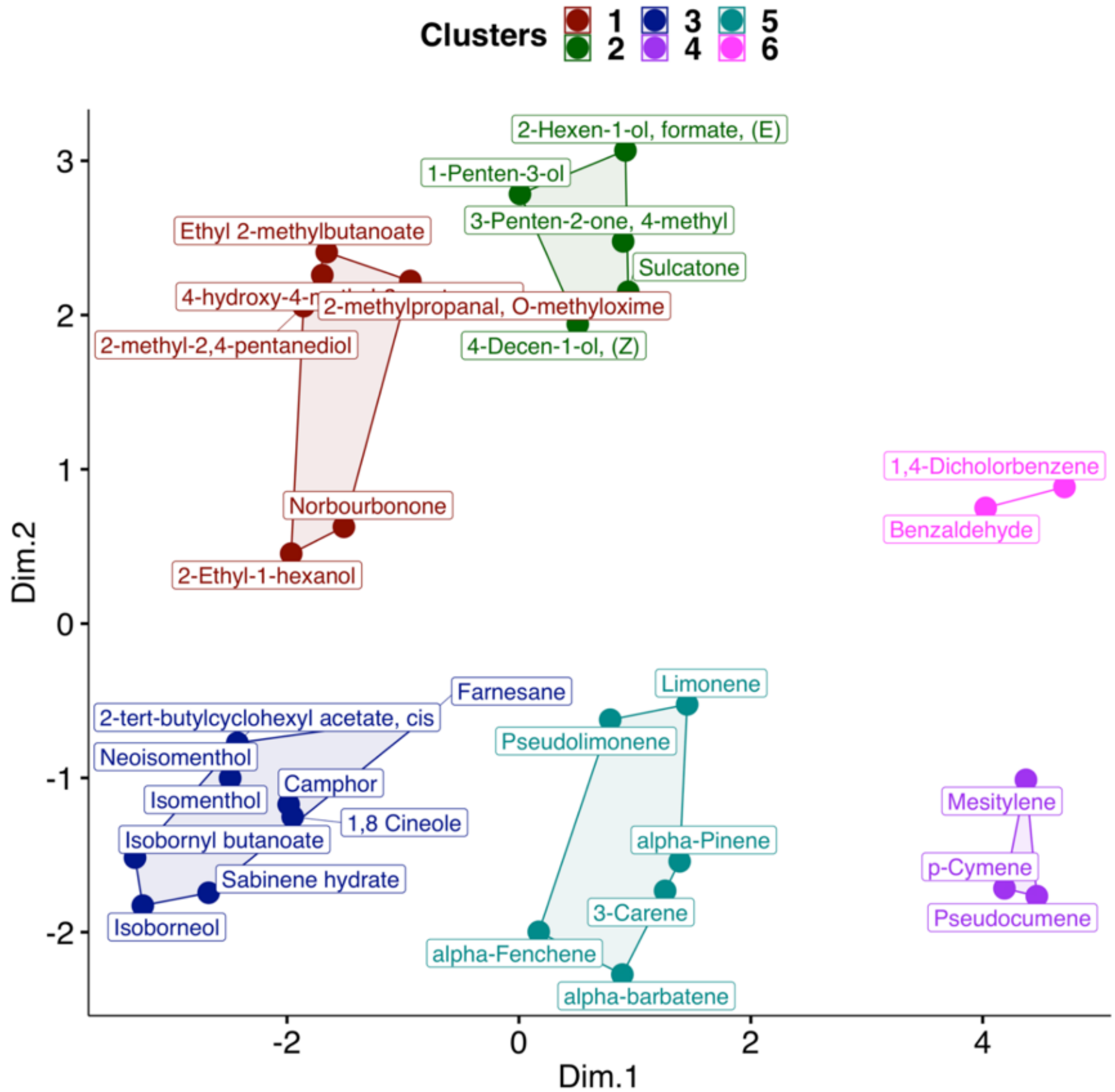

Figure S3. K-means clustering of chemicals based on their atomic structure, plotted along two principal dimensions from multidimensional scaling (MDS). Chemicals are color-coded by cluster: Cluster 1 (red) includes oxygenated and branched hydrocarbons like Ethyl 2-methylbutanoate; Cluster 2 (green) contains unsaturated alcohols and ketones such as 2-Hexen-1-ol, formate (E); Cluster 3 (blue) features cyclic alcohols and terpenoids like Isoborneol; Cluster 4 (purple) includes methyl-substituted aromatics such as Mesitylene; Cluster 5 (cyan) highlights monoterpenes like Limonene and alpha-Pinene; and Cluster 6 (pink) groups aromatic chemicals like Benzaldehyde. Cluster separation reflects structural similarities, with proximity indicating chemical relationships within the principal dimensions.

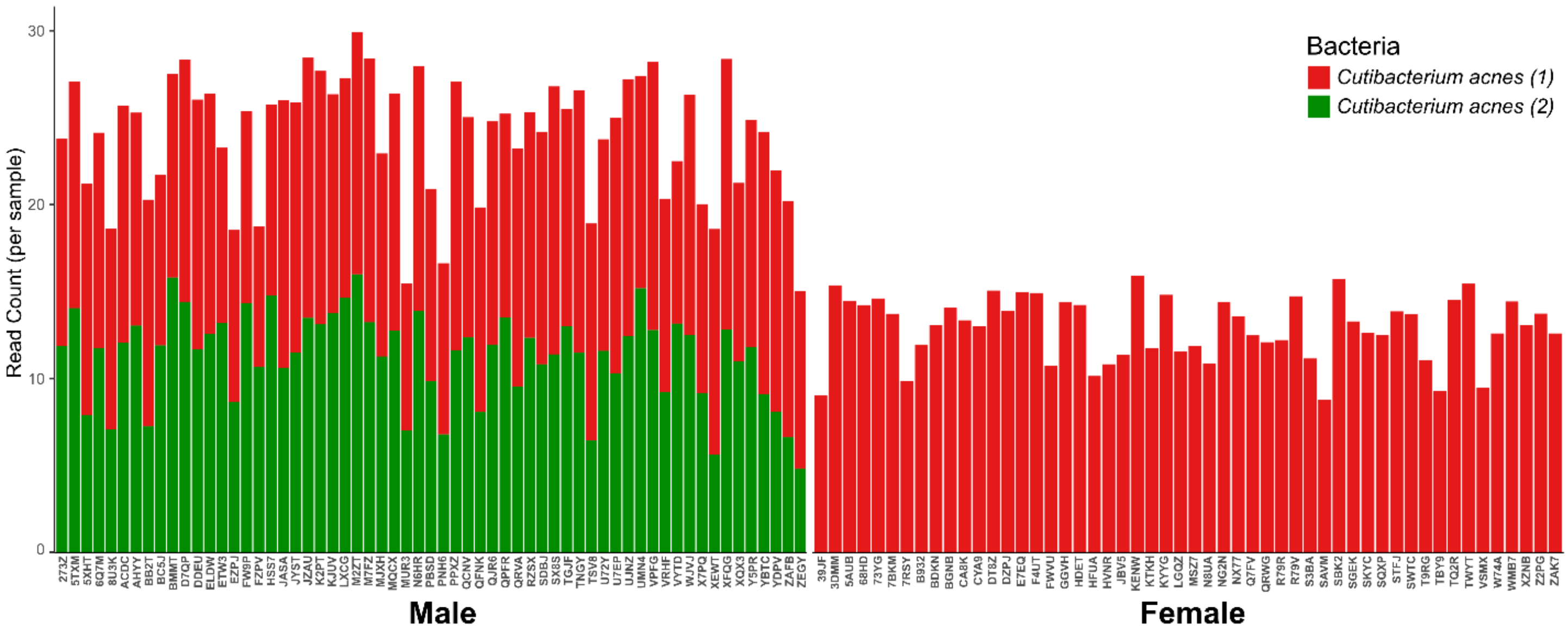

Figure S4. Core microbiome of all bacteria detected for each self-reported male (left) and female (right) individual. Two amplicon sequence variants (ASVs) of *C. acnes* comprise the male core microbiome, while one ASV of *C. acnes* comprises the female core microbiome. All bacteria were normalized and scaled using cumulative sum scaling.

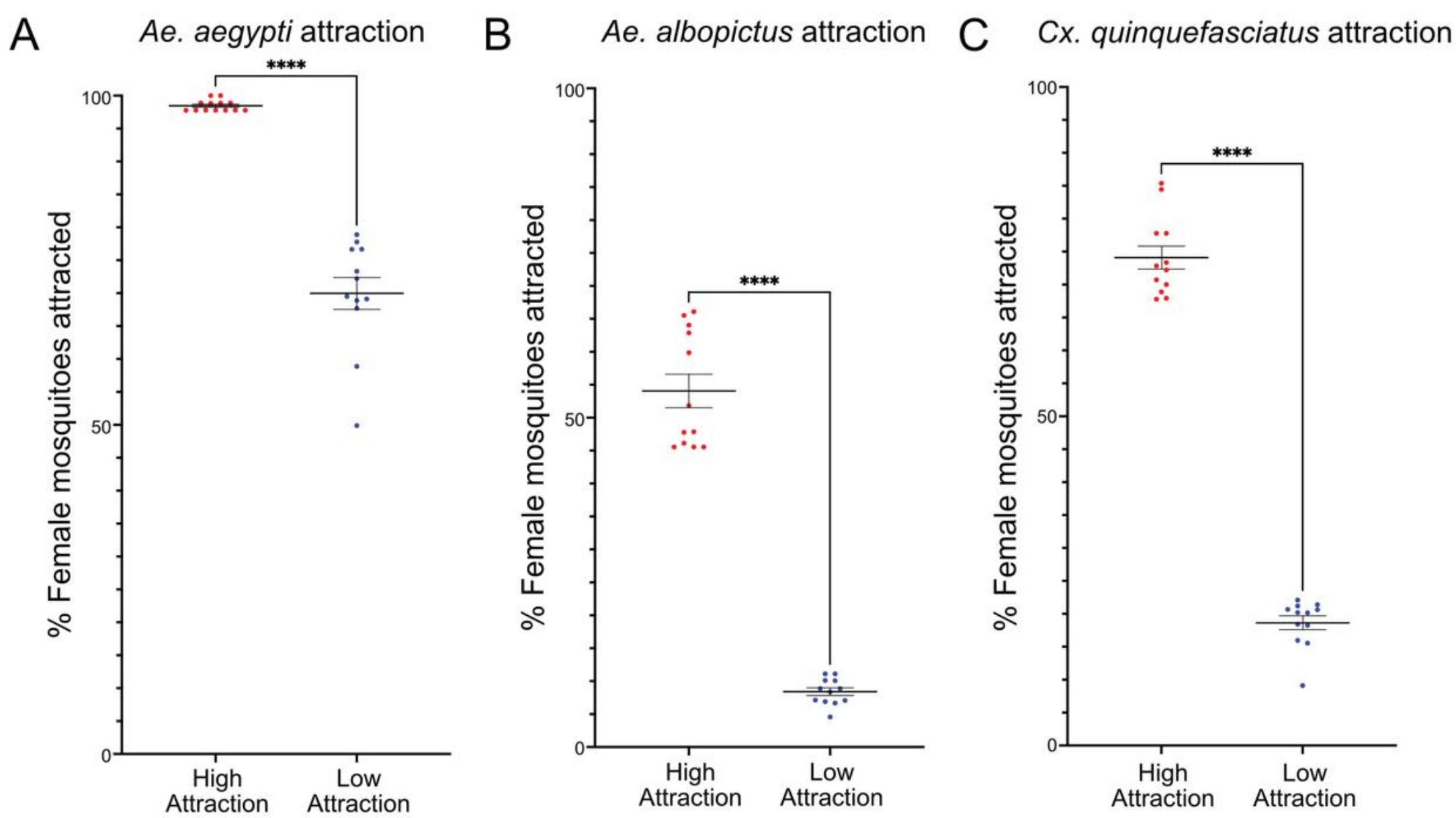

Figure S5. (A) Wilcoxon rank-sum test comparing *Ae. aegypti* high and low-attraction groups. ($p < 0.001$). **(B)** Wilcoxon rank-sum test comparing *Ae. albopictus* high and low attraction groups. ($p < 0.001$). **(C)** Wilcoxon rank-sum test comparing *Cx. quinquefasciatus* high and low attraction groups. ($p < 0.001$). For all tests, high attraction dots are in red and low attraction dots are in blue. Error bars show standard error of the mean.

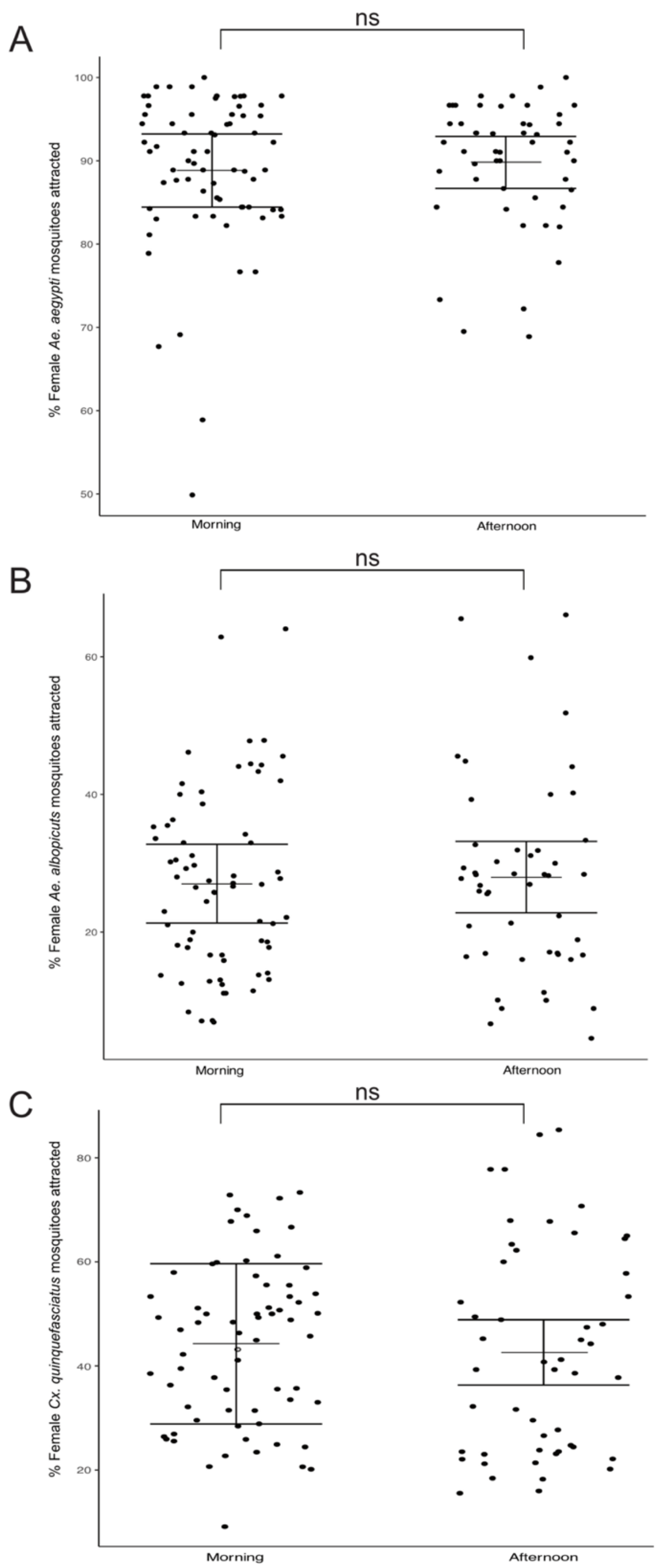

Figure S6. Participants tested in the morning or afternoon evaluated by **(A)** *Ae. aegypti,* **(B)** *Ae. albopictus,* or **(C)** *Cx. quinquefasciatus* attraction. A Wilcoxon test showed attraction is not significantly different for *Ae. aegypti* ($P = 0.7$), *Ae. albopictus* ($P = 0.81$), or *Cx. quinquefasciatus* ($P = 0.33$). Errors bars show standard deviation.

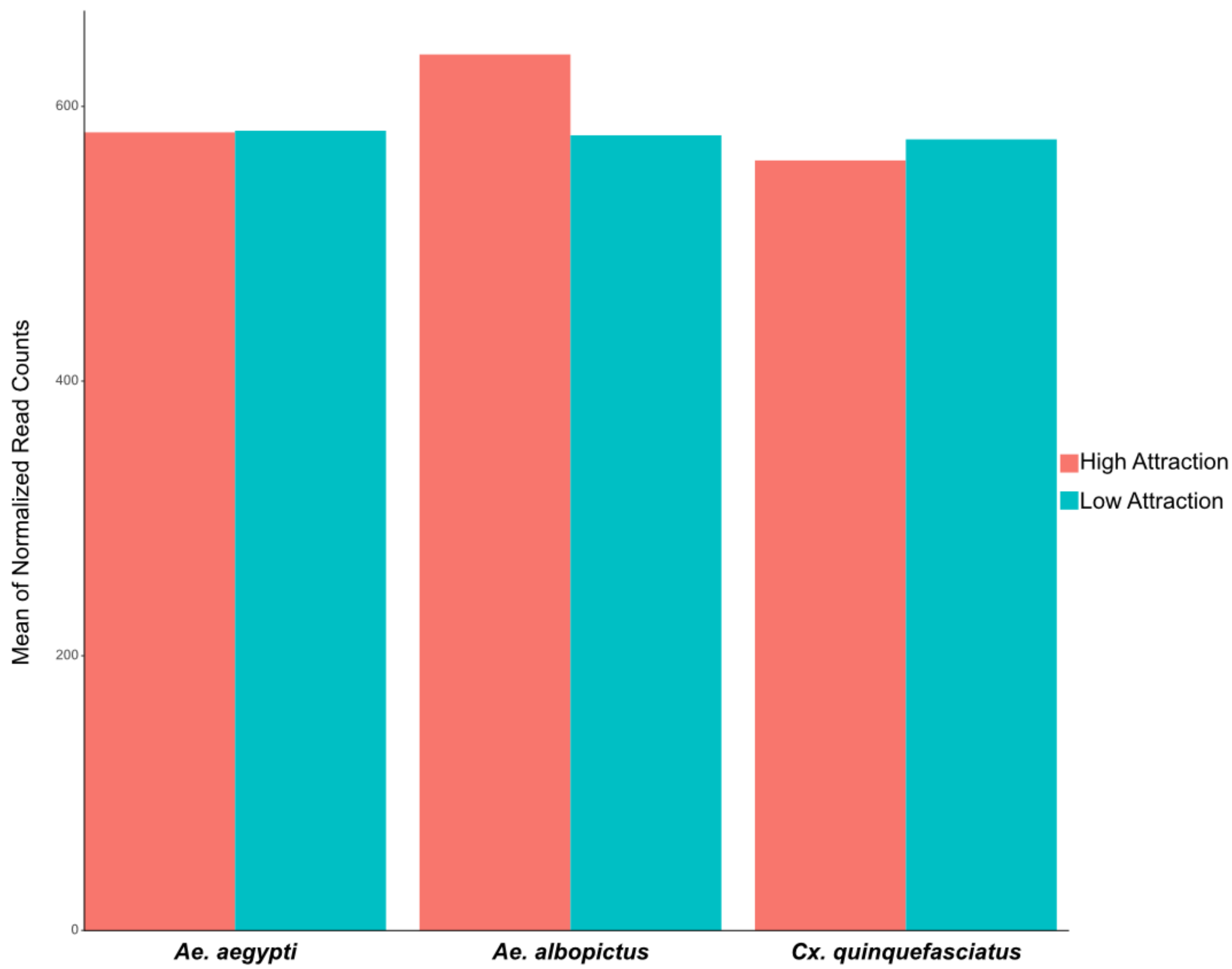

Figure S7. Mean normalized read counts of human skin microbiome for high and low attraction groups in each mosquito species. *Ae. aegypti* high attraction had an average of 581.1 and low attraction had an average of 582.3 *Ae. albopictus* had a high attraction average of 637.8 and a low attraction average of 578.9. *Cx. quinquefasciatus* high attraction had an average of 560.7 and low attraction had an average of 576.0.

Covariate	Mosquito species	p-value
**Sex (Male)**	*Ae. aegypti*	**0.02371**
**Age**	*Ae. aegypti*	0.49564
**Temperature**	*Ae. aegypti*	0.06339
**Race (Black)**	*Ae. aegypti*	**0.0191**
**Race (Hispanic)**	*Ae. aegypti*	0.33307
**Race(Mixed)**	*Ae. aegypti*	**0.00877**
**Race(White)**	*Ae. aegypti*	**0.00698**
**Sex (Male)**	*Ae. albopcitus*	0.864
**Age**	*Ae. albopcitus*	0.689
**Temperature**	*Ae. albopcitus*	0.21
**Race (Black)**	*Ae. albopcitus*	0.29
**Race (Hispanic)**	*Ae. albopcitus*	0.92
**Race(Mixed)**	*Ae. albopcitus*	0.208
**Race(White)**	*Ae. albopcitus*	0.459
**Sex (Male)**	*Cx. quinquefasciatus*	0.2234
**Age**	*Cx. quinquefasciatus*	0.3175
**Temperature**	*Cx. quinquefasciatus*	0.4502
**Race (Black)**	*Cx. quinquefasciatus*	0.0649
**Race (Hispanic)**	*Cx. quinquefasciatus*	0.1451
**Race(Mixed)**	*Cx. quinquefasciatus*	0.3799
**Race(White)**	*Cx. quinquefasciatus*	0.2096

Table S1. A linear mixed model was constructed for each species to assess the effect of demographics on mosquito attraction. Tested variables included sex, age, and race, as well as temperature. Ethnicity data was removed due to high VIF (>10). Bold p-values indicate significance.

Comparison	Cohen's d value	Post-hoc effect size interpretation (Cohen, 1988)
Asian / Black	0.806	Large
Asian / Hispanic	0.59	Medium
Asian / Mixed	0.87	Large
Asian / White	0.78	Large
Black / Hispanic	0.31	Small
Black / Mixed	0.11	Small
Black / White	0.0073	Small
Hispanic / Mixed	0.42	Medium
Hispanic / White	0.29	Small
Mixed / White	0.11	Small
Male / Female	0.5066	Medium

Table S2. Cohen's d values for demographic comparisons for *Ae. aegypti* mosquito attraction. Values are interpreted using post-hoc effect size interpretations from Cohen, 1988 and Rosnow & Rosenthal, 1996 (52, 53).

Odor	*Ae. aegypti*	*Ae. albopictus*	*Cx. quinquefasciatus*
Unknown (RI 811)	High Attraction ▲	High Attraction ▲	Low Attraction ▲
Ethyl 2-methylbutanoate	High Attraction ▲	High Attraction ▲	High Attraction ▲
(E)-2-Hexen-1-ol, formate	High Attraction ▲	High Attraction ▲	High Attraction ▲
alpha-Pinene	High Attraction ▲	High Attraction ▲	High Attraction ▲
Benzaldehyde	High Attraction ▲	High Attraction ▲	Low Attraction ▲
Pseudolimonene	High Attraction ▲	Low Attraction ▲	High Attraction ▲
2-Ethyl-1-hexanol	High Attraction ▲	Low Attraction ▲	High Attraction ▲
1,4-Dichlorobenzene	High Attraction ▲	High Attraction ▲	Low Attraction ▲
Isoborneol	High Attraction ▲	High Attraction ▲	Low Attraction ▲
Farnesane	High Attraction ▲	High Attraction ▲	Low Attraction ▲
1-Penten-3-ol	Low Attraction ▲	Low Attraction ▲	Low Attraction ▲
4-methyl-3-Penten-2-one	Low Attraction ▲	High Attraction ▲	Low Attraction ▲
Unknown (RI 834)	Low Attraction ▲	High Attraction ▲	Low Attraction ▲
4-Hydroxy-4-methyl-2-pentanone	Low Attraction ▲	Low Attraction ▲	Low Attraction ▲
2-Methyl-2,4-pentanediol	Low Attraction ▲	High Attraction ▲	Low Attraction ▲
2-Methylpropanal, O-methyloxime	Low Attraction ▲	Low Attraction ▲	Low Attraction ▲
alpha-Fenchene	Low Attraction ▲	High Attraction ▲	High Attraction ▲
Mesitylene	Low Attraction ▲	High Attraction ▲	High Attraction ▲
Pseudocumene	Low Attraction ▲	High Attraction ▲	Low Attraction ▲
Sulcatone	Low Attraction ▲	High Attraction ▲	Low Attraction ▲
3-Carene	Low Attraction ▲	Low Attraction ▲	Low Attraction ▲
p-Cymene	Low Attraction ▲	High Attraction ▲	High Attraction ▲
Limonene	Low Attraction ▲	High Attraction ▲	Low Attraction ▲
1,8-Cineole	Low Attraction ▲	High Attraction ▲	Low Attraction ▲
Unknown (RI 1073)	Low Attraction ▲	High Attraction ▲	Low Attraction ▲
Sabinene hydrate	Low Attraction ▲	High Attraction ▲	High Attraction ▲
Camphor	Low Attraction ▲	High Attraction ▲	Low Attraction ▲
Isomenthol	Low Attraction ▲	Low Attraction ▲	Low Attraction ▲
Neoisomenthol	Low Attraction ▲	High Attraction ▲	Low Attraction ▲
(Z)-4-Decen-1-ol	Low Attraction ▲	High Attraction ▲	High Attraction ▲
Unknown (RI 1301)	Low Attraction ▲	High Attraction ▲	High Attraction ▲
cis-2-tert-Butylcyclohexyl acetate	Low Attraction ▲	High Attraction ▲	Low Attraction ▲
Unknown (RI 1344)	Low Attraction ▲	High Attraction ▲	Low Attraction ▲
alpha-Barbatene	Low Attraction ▲	High Attraction ▲	High Attraction ▲
Isobornyl butanoate	Low Attraction ▲	High Attraction ▲	High Attraction ▲
Norbourbonone	Low Attraction ▲	High Attraction ▲	Low Attraction ▲

Table S3. Volatile organic compounds were identified via GC-MS and evaluated for greater prevalence in high attraction or low attractions groups for each mosquito species. Text in red denotes that the odor was more abundant in the high attraction group for that mosquito species. Text in blue denotes that the odor was more abundant in the low attraction group for that mosquito species.
